# Supplementary figures and images for: Effect of foot reflexology on chronic pain in Parkinson’s disease: A randomized controlled trial
Source: PLoS One. 2025 Jul 28;20(7):e0327865. doi: 10.1371/journal.pone.0327865 (PMC12303304; doi:10.1371/journal.pone.0327865)

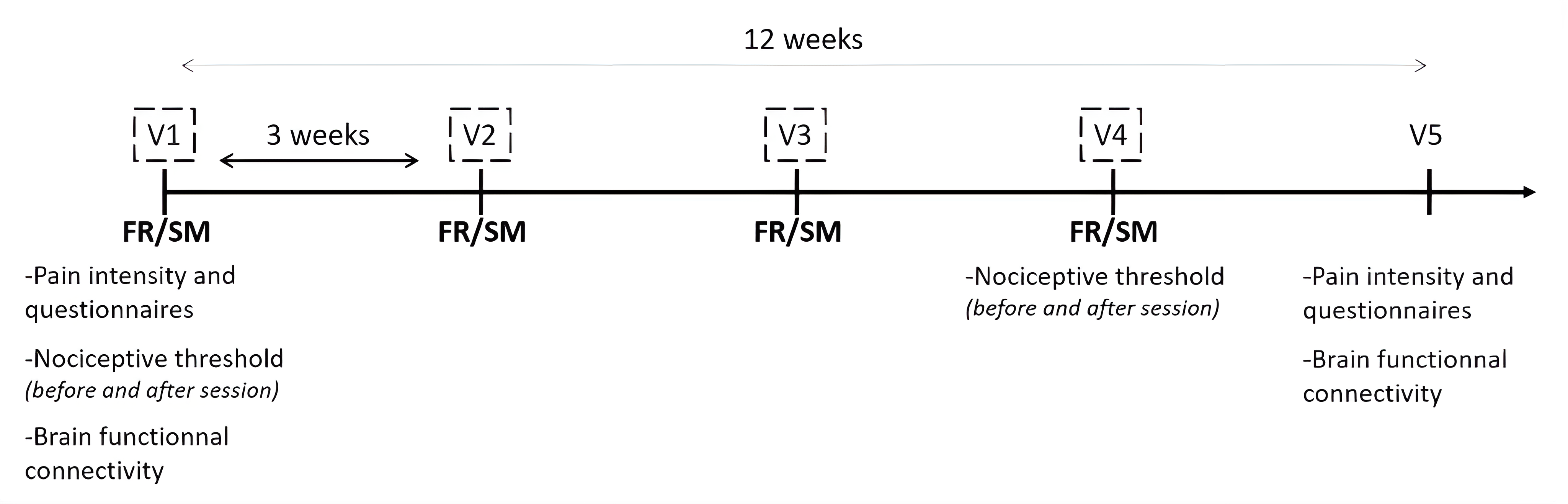

Supplement: S1 Fig — V: Visit, FR: Foot Reflexology, SM: Sham Massage. (TIFF) [file pone.0327865.s001.tiff]

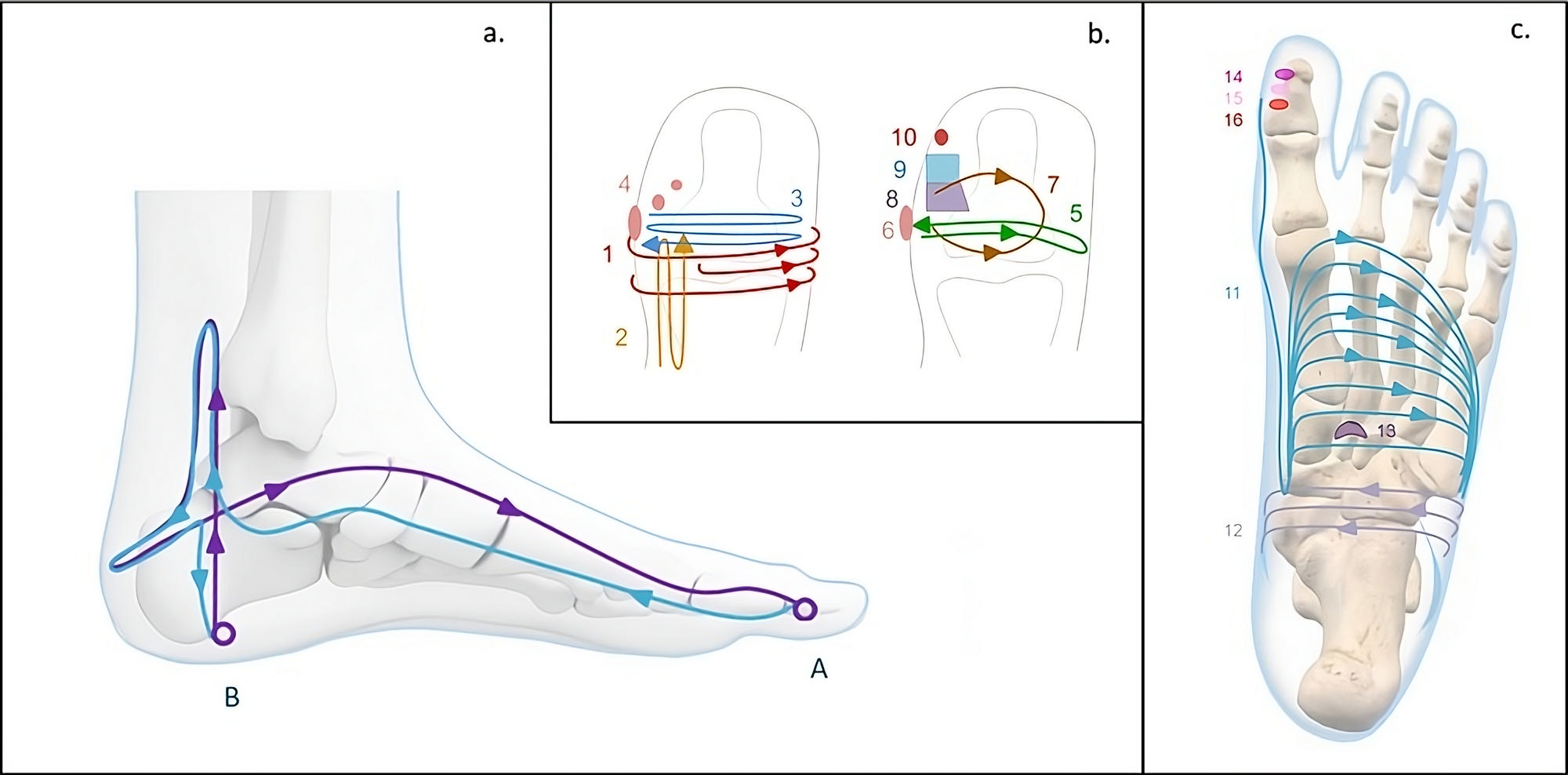

Supplement: S2 Fig — Internal arch of left foot view, b. Big toe, plantar arch of left foot view, c. Plantar arch of left foot view. A. Base of distal phalange of large toe = C1 vertebra reflex, B. Medial process of calcaneus = coccyx reflex, 1. occipito-cervical junction reflex, 2. brainstem reflex, 3. 4th ventricle and cerebellum reflex, 4. sphenobasilar symphysis, pituitary, hypothalamus reflex, 5. tent of cerebellum reflex, 6. sphenobasilar symphysis reflex, 7. hippocampus reflex, 8. septum pellucidum reflex, 9. corpus callosum reflex, 10. epiphysis reflex, 11. diaphragm reflex, 12. diaphragm insertion reflex, 13. adrenals reflex, 14. epiphysis reflex, 15. hypothalamus reflex, 16. pituitary reflex. FR circuit: 1) Starting with the left foot, then the right foot. Relaxation movement: complete smoothing of foot x3, ankle smoothing x3. 2) Starting with the spine reflex: the pathway followed is from point A to point B (in blue), then from point B to point A (in purple), see S2 Fig a. 3) Starting with the left foot, then the right foot. Then, the 10 reflex zones associated with the brain reflex, as shown in S2 Fig b, zone 1 to zone 10, are successively stimulated on the big toe, with strong stimulation in the upper (locus niger) and middle (raphe nuclei) parts. Next, the beginning of spine circuit from the point A to the coccyx reflex (point B), shown in blue in the S2 Fig a, and the coccyx reflex (point B) is pump 3x (pressure/ release). 4) Starting with the left foot, then the right foot. The emotional circuit is stimulated, starting with the diaphragm reflex (zone 11), 9 turns around the foot, then 3 turns around the foot in front of the diaphragm insertion reflex (zone 12) (see S2 Fig c). The stress axis zones are stimulated simultaneously: adrenal reflex (point 13), epiphysis, hypothalamus, and pituitary (point 14, 15, 16 respectively, see S2 Fig c). 5) Ending with the beginning of spine circuit to coccyx reflex (point B), shown in blue in S2 Fig a, then the coccyx reflex (point B) [file pone.0327865.s002.tif]

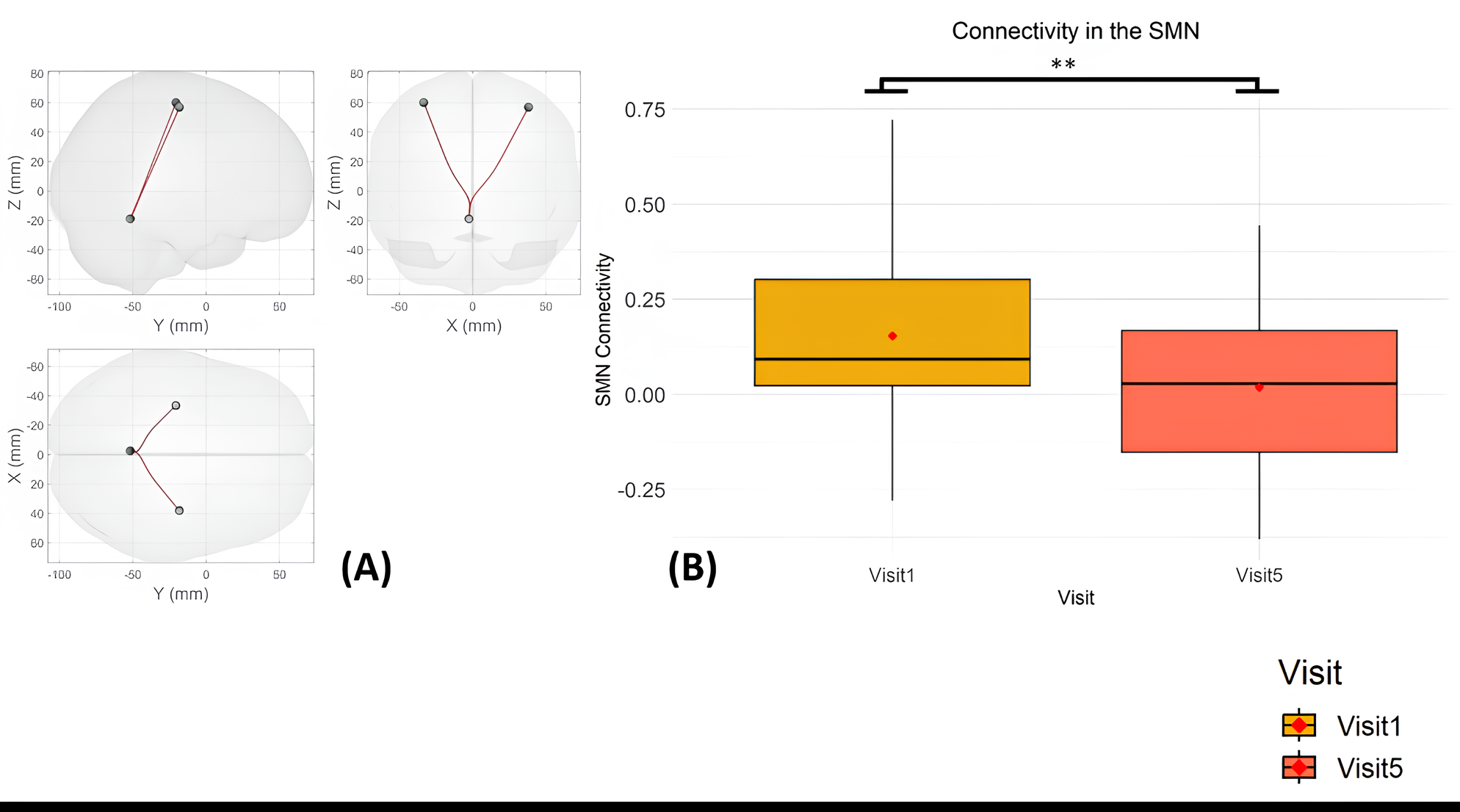

Supplement: S3 Fig — A) Representation of the clusters on a 3D brain. Cluster +02 + 16–10 is formed of 255 voxels with 170 voxels covering 15% of the subcallosal cortex, 9 voxels covering 11% of the right nucleus accumbens and 3 voxels covering a non-labelled area. Size p-FDR = 0.000678, size p-unc = 0.000029. Cluster +02 + 52–26 is formed of 137 voxels, with 83 voxels covering 8% of the frontal medial cortex, 43 voxels covering 1% of the right frontal pole and 1 voxel covering less than 1% of the left frontal pole. Size p-FDR = 0.011661, size p-unc = 0.001014. B) Boxplots of the connectivity between the left nucleus accumbens and cluster +02 + 16–10 according to visits and groups. There was no significant difference at baseline (p-value = 0.14). After intervention there was a significant decrease in the FR group (mean at V1: 0.249 ± 0.094, mean at V5: 0.130 ± 0.101, difference: −0.119 ± 0.097, p < 0.001) and a significant increase in the SM group (mean at V1: 0.156 ± 0.113, mean at V5: 0.287 ± 0.107, difference: 0.131 ± 0.066, p < 0.001). C) Boxplots of the connectivity between the left nucleus accumbens and cluster +02 + 52–26 according to visits and groups. There was a significant difference at baseline of 0.140, SEM: 0.042 (p-value = 0.01). After intervention there was a significant decrease in the FR group (mean at V1: 0.122 ± 0.110, mean at V5: −0.020 ± 0.114, difference: −0.142 ± 0.109, p < 0.001) and a significant increase in the SM group (mean at V1: −0.018 ± 0.116, mean at V5: 0.086 ± 0.104, difference: 0.104 ± 0.145, p-value = 0.002). Blue= Foot Reflexology (FR); light blue= Sham Massage (SM). (TIFF) [file pone.0327865.s003.tiff]
