# Supplementary material for: Effect of foot reflexology on chronic pain in Parkinson’s disease: A randomized controlled trial
Source: PLoS One. 2025 Jul 28;20(7):e0327865. doi: 10.1371/journal.pone.0327865 (PMC12303304; doi:10.1371/journal.pone.0327865)
Supplement: S1 Table — Means ± standard deviations; MoCA: Montreal Cognitive Assessment; LEDD: levodopa equivalent daily dose; MDS-UPDRS III: Movement Disorder Society – Unified Parkinson’s Disease Rating Scale part III; VAS: Visual Analogue Scale; KPPS: King’s Parkinson Pain Scale; BPI: Brief Pain Inventory; SF-MPQ: Short Form McGill Pain Questionnaire; CPAQ-8: Chronic Pain Acceptance Questionnaire 8; HAD: Hospital Anxiety and Depression. For all variables, there was no statistical difference between groups p > 0.05. (DOCX) [file pone.0327865.s005.docx]

**S1 Table. Demographic and clinical data at baseline for responders and non-responders**

|  | Population | | | Responders | | | Non responders | | | Difference between groups p-value |
| --- | --- | --- | --- | --- | --- | --- | --- | --- | --- | --- |
| **N** | 30 | | | 14 | | | 16 | | |  |
| **Gender (women)** | 18 | | | 7 | | | 11 | | | 0.30 |
| **Age (years)** | 65.8 | ± | 7.41 | 64.7 | ± | 6.01 | 66.8 | ± | 8.53 | 0.49 |
| **Disease duration (years)** | 7.37 | ± | 5.22 | 7.14 | ± | 4.37 | 7.56 | ± | 6.00 | 0.63 |
| **Pain duration (years)** | 9.03 | ± | 8.69 | 6.57 | ± | 3.44 | 11.2 | ± | 11.2 | 0.49 |
| **MoCA** | 27.9 | ± | 1.80 | 28.0 | ± | 1.66 | 27.9 | ± | 1.96 | 1.0 |
| **LEDD (mg/day)** | 721 | ± | 423 | 758 | ± | 528 | 689 | ± | 320 | 0.90 |
| **MDS-UPDRS III** | 25.1 | ± | 15.8 | 27.1 | ± | 16.0 | 23.3 | ± | 15.9 | 0.45 |
| **mean VAS** | 56.0 | ± | 14.3 | 58.6 | ± | 15.1 | 53.7 | ± | 13.7 | 0.38 |
| **max VAS** | 73.7 | ± | 16.0 | 76.4 | ± | 19.7 | 71.4 | ± | 12.1 | 0.39 |
| **KPPS** | 24.5 | ± | 17,3 | 23.9 | ± | 21.2 | 24.9 | ± | 13.8 | 0.30 |
| **BPI** | 30.3 | ± | 11.7 | 29.6 | ± | 14.2 | 30.9 | ± | 9.47 | 0.42 |
| **SF-MPQ total** | 16.7 | ± | 8.30 | 17.4 | ± | 7.69 | 16.0 | ± | 9.00 | 0.66 |
| **SF-MPQ sensory** | 12.1 | ± | 5.95 | 13.0 | ± | 4.62 | 11.3 | ± | 6.95 | 0.32 |
| **SF-MPQ affective** | 4.60 | ± | 3.16 | 4.43 | ± | 3.65 | 4.75 | ± | 2.77 | 0.63 |
| **CPAQ-8 total** | 26.7 | ± | 7.63 | 26.4 | ± | 7.23 | 27.0 | ± | 8.19 | 0.88 |
| **CPAQ-8 willingness** | 9.93 | ± | 4.99 | 9.86 | ± | 5.02 | 10.0 | ± | 5.13 | 0.85 |
| **CPAQ-8 activity engagement** | 16.8 | ± | 5.03 | 16.6 | ± | 5.93 | 17.0 | ± | 4.27 | 0.85 |
| **HAD total** | 16.0 | ± | 6.11 | 15.9 | ± | 7.23 | 16.1 | ± | 5.13 | 0.95 |
| **HAD Anxiety** | 9.34 | ± | 3.82 | 8.93 | ± | 4.34 | 9.73 | ± | 3.37 | 0.60 |
| **HAD Depression** | 6.69 | ± | 3.63 | 7.00 | ± | 3.68 | 6.40 | ± | 3.68 | 0.66 |
| **Nociceptive threshold** | 41.8 | ± | 2.49 | 42.5 | ± | 2.71 | 41.1 | ± | 2.17 | 0.10 |
| **Aetiology of pain** |  |  |  |  |  |  |  |  |  | 0.70 |
| **Musculoskeletal** | 9 (30%) | |  | 4 |  |  | 5 |  |  |  |
| **Radicular** | 1 (3.3%) | |  | 0 |  |  | 1 |  |  |  |
| **Restless Leg Syndrome** | 1 (3.3%) | |  | 1 |  |  | 0 |  |  |  |
| **Dystonic** | 1 (3.3%) | |  | 0 |  |  | 1 |  |  |  |
| **Central** | 13 (43.3%) | |  | 7 |  |  | 6 |  |  |  |
| **Others** | 5 (16.7%) | |  | 2 |  |  | 3 |  |  |  |

Means ± standard deviations; MoCA: Montreal Cognitive Assessment; LEDD: levodopa equivalent daily dose; MDS-UPDRS III: Movement Disorder Society – Unified Parkinson’s Disease Rating Scale part III; VAS: Visual Analogue Scale; KPPS: King’s Parkinson Pain Scale; BPI: Brief Pain Inventory; SF-MPQ: Short Form McGill Pain Questionnaire; CPAQ-8: Chronic Pain Acceptance Questionnaire 8; HAD: Hospital Anxiety and Depression. For all variables, there was no statistical difference between groups p>0.05.
