# Supplementary material for: Effect of foot reflexology on chronic pain in Parkinson’s disease: A randomized controlled trial
Source: PLoS One. 2025 Jul 28;20(7):e0327865. doi: 10.1371/journal.pone.0327865 (PMC12303304; doi:10.1371/journal.pone.0327865)
Supplement: S1 Protocol — (DOCX) [file pone.0327865.s008.docx]

**Exploratory study of the impact of foot reflexology on chronic pain in Parkinson's disease patients.**

**DOREPAR**

**RC 31/20/0279**

**INTERVENTION RESEARCH PROTOCOL**

**INVOLVING THE HUMAN PERSON**

***(Category 2 with minimal risks and constraints)***

Version n°2.0 dated 02/09/2021

RCB-ID Number: 2020-A03036-33

**This interventional research has obtained funding from the France Parkinson association**

Proponent :

*CHU TOULOUSE – Hôtel Dieu – 2, rue Viguerie – TSA 80035*

*31059 Toulouse cedex 9*

Principal investigator (single-center research):

Dr Christine Brefel-Courbon

Pierre Paul Riquet Hospital

1 place du docteur Baylac

TSA 400 31

31059 Toulouse cedex 9

**This protocol was designed and drafted based on version 5.0 of 30/10/2017 of the GIRCI SOHO model protocol**

# PROTOCOL UPDATE HISTORY

| **VERSION** | **DATE** | **REASON FOR UPDATE** |
| --- | --- | --- |
| 1 | 15/07/2020 | Version submitted for promotion request |
| 1.1 | 19/11/2020 | Version submitted to the CPP |
| 1.2 | 30/12/2020 | Version in response to the CPP Sud-Méditerannée |
| 1.3 | 18/02/2021 | Version in response to the CPP Sud-Méditerannée (methodologist) |
| 2.0 | 02/09/2021 | Substantial Modification Version No. 1 |

**Protocol Signature Page**

**Exploratory study of the impact of foot reflexology on chronic pain in Parkinson's patients.**

**DOREPAR**

**Sponsor Code: RC31/20/0279**

| **Promoter**    TOULOUSE University Hospital  Hôtel Dieu  2, rue Viguerie TSA 80035  31059 TOULOUSE cedex 9 | Done in Toulouse,    The | Prof. Olivier LAIREZ,  Director of Research and Innovation signature |
| --- | --- | --- |
| **Principal investigator**  Dr Christine Brefel-Courbon  Pierre Paul Riquet Hospital  1 place du docteur Baylac  TSA 400 31  31059 Toulouse cedex 9 | Done at    The | Dr Christine Brief-  Courbon  Neurology Department    *signature* |
| **Scientific Manager**  Emeline Descamps  UMR1214 - Inserm/UPS – ToNIC  (Toulouse NeuroImaging Center)  Baudot Pavilion, Purpan University Hospital  31059 Toulouse – Cedex 3 | Done at    The | *Emeline Descamps signature* |

**MAIN CORRESPONDENTS**

**Coordinating Investigator/Principal**

Dr Christine Brefel-Courbon

Pierre Paul Riquet Hospital

1 PLACE DU DOCTEUR BAYLAC

TSA 400 31

31059 TOULOUSE CEDEX 9

## Scientific Manager

Emeline Descamps, PhD

CNRS Research Fellow

Toulouse NeuroImaging Centre (ToNIC –

UMR1214 Inserm/UPS)

Baudot Pavilion, Purpan University Hospital

31059 Toulouse – Cedex 3 Tel:

**Clinical Research Vigilance Unit**

Dr Pascale OLIVIER-ABBAL

Medical and Clinical Pharmacology Department

& Direction from the Research of the

Development and Innovation

TOULOUSE UNIVERSITY HOSPITAL

## Project Management Assistance

Estelle Harroch-Project Manager

Steering Assistance

CEP - Centre Expert Parkinson

Toulouse Purpan University Hospital

Pierre Paul RIQUET Building

Department of Neurology

Place du Dr BAYLAC

TSA 40031 – 31059 TOULOUSE Cedex 9 **Developer**

CHU TOULOUSE – Hôtel Dieu

2, rue Viguerie – TSA 80035

31059 Toulouse cedex 9

Regulatory CDP: Nadège ALGANS

**Centre for Methodology and Management of Biological Strategies**

**data**

*Methodologist:* Agnès Sommet

*Statistician:* Vanessa Rousseau Phd -

Biostatistician

Medical and Clinical Pharmacology Department -

Toulouse University Hospital

University of Toulouse - Faculty of Medicine

Center from Pharmacovigilance, from

Pharmacoepidemiology and Information on

Medicine

Equipe MéDatAS - CIC CHU Toulouse - CIC 1436

*Imaging:* Toulouse NeuroImaging Centre

(ToNIC – Inserm/UPS UMR1214)

CHU PURPAN - Baudot Pavilion

**Randomization**

Christophe Morin

MeDatAS-CIC Unit

CIC 1436 Toulouse University Hospital/UPS/Inserm

**SUMMARY**

DOREPAR

Version No.

2.0

of the

02

/

09

/2021

establishment

1. RESEARCH SUMMARY 8

**ABSTRACT 11**

1. SCIENTIFIC RATIONALE AND GENERAL DESCRIPTION 14
   1. *Current state of knowledge 14*
   2. *Research hypotheses and expected results 16*
   3. *Justification of methodological choices 16*
   4. *Benefit/risk ratio 17 2.5. Expected benefits 17*

*2.6.*  *Rationale for low level of intervention 17*

1. RESEARCH OBJECTIVES 19 *3.1. Main objective 19*

*3.2.*  *Secondary Objectives 19*

1. JUDGING CRITERIA 20
   1. *Primary outcome 20*
   2. *Secondary outcomes 20*
2. RESEARCH DESIGN 22 *5.1. Research Outline 22*

*5.2.*  *Methods for Randomization 22*

1. CRITERARIESELIGIBILITY 24
   1. *Inclusion criteria 24 6.2. Non-inclusion criteria 24*

*6.3.*  *Feasibility and Recruitment Procedures 24*

1. TREATMENT(S)/STRATEGY(S)/PROCEDURE(S) RESEARCH 25
   1. *Investigational Treatment/Strategy/Procedure 25*
   2. *Treatment/Strategy/Comparison Procedure 28*
2. ASSOCIATED TREATMENTS AND PROCEDURES(E)S 29
   1. *Authorized Associated Treatments/Procedures 29 8.2.*  *Prohibited Associated Treatments/Procedures 29*
3. PROGRESS OF RESEARCH 30 *9.1. Timeline of the search 30*

*9.2. Summary table of participant monitoring 30 9.3. Pre-inclusion/inclusion visit 31 9.4. Visit/Randomization Process 31*

- 1. *Follow-up visits 32*
  2. *Completion visit of the research 32*
  3. *Rules for stopping a person's participation in research 32*
  4. *Research constraints and possible compensation for participants 33*

**VIGILANCE PART FOR CATEGORY 2 SEARCHES:** ERROR! BOOKMARK UNDEFINED.

1. ADVERSE EVENT MANAGEMENT / ADVERSE REACTIONS / INCIDENTS 33
2. STATISTICAL ASPECTS 33
   1. *Calculating the study size 33*
   2. *Statistical methods used 34*
   3. *Security analysis* ***Error! Bookmark not set.***
3. RESEARCH OVERSIGHT 36

Page 5 of 71

DOREPAR

Version No.

2.0

of the

02

/

09

/2021

establishment

13.

D

KINGS

D

’

ACCESS TO DATA AND

DOCUMENTS SOURCE

36

- 1. *Access to data 36*
  2. *Source data 36*
  3. *Data Privacy 36*

1. QUALITY CONTROL AND ASSURANCE 37
   1. *Guidelines for data collection 37*
   2. *Quality control 37*
   3. *Data Management 37*
   4. *Audit and inspection 38*
2. ETHICAL AND REGULATORY CONSIDERATIONS 39
3. RETENTION OF RESEARCH DOCUMENTS AND DATA 41
4. **FINAL REPORT 41**
5. RULES RELATING TO PUBLICATION 42 *18.1. Scientific communications 42*
   1. *Communication of results to participants 42*
   2. *Data transfer 42*

**BIBLIOGRAPHICAL REFERENCES 43**

**ANNEXES 45**

Page 6 of 71

**LIST OF ABBREVIATIONS**

| ANSM | National Agency for the Safety of Medicines and Health Products |
| --- | --- |
| CEP | Centre Expert Parkinson |
| CPP | Committee for the Protection of Persons |
| EvI | Adverse Event |
| EvIG | Serious Adverse Event |
| EIG | Serious adverse reaction |
| EIGI | Unexpected serious adverse reaction |
| SUSAR | Suspected Unexpected Serious Adverse Reaction |
| FR | Foot Reflexology |
| SM | Sham Massage |
| KPPS | King's Parkinson's disease Pain Scale |
| BPI | Brief Pain Inventory |
| VAS | Visual Analogue Scale |
| 3PDQ | Primary Parkinsonian Pain Diagnosis Questionnaire |
| NMI | Non-drug intervention |
| MRI | Magnetic Resonance Imaging |
| CPAQ-8 | Chronic Pain Acceptance Questionnaire 8 |
| HAD | Hospital Anxiety and Depression Scale |

## 1. RESEARCH SUMMARY

| **PROMOTER** | Toulouse University Hospital |
| --- | --- |
| **PRINCIPAL INVESTIGATOR** | Dr Christine Brefel-Courbon |
| **TITLE** | Exploratory study of the impact of foot reflexology on chronic pain in Parkinson's patients. DOREPAR |
| **JUSTIFICATION/**  **CONTEXT** | At present, drugs are not effective enough to relieve chronic pain in Parkinson's disease (PD). This encourages caregivers to explore new therapeutic strategies such as non-drug interventions (NPIs). This project is innovative and original from two points of view: on the one hand, the pain of patients in PD is still often underestimated, there is in the simple fact of devoting a study to it a real dynamic of change in the consideration of the patients concerned and in its management. On the other hand, the increasing use of NMIs requires rigorous dedicated clinical studies. However, currently, it is clear that very few good quality studies have been carried out on the subject. In this research, we propose to scientifically and rigorously evaluate foot reflexology (FR) in order to reach reliable conclusions in chronic pain in Parkinson's patients. The challenge is to provide concrete help to Parkinson's pain patients and to enable doctors to recommend this type of care in an informed manner. |
| **OBJECTIVES** | **The main objective** is to evaluate the variation in the average intensity of chronic pain by PR measured by a Visual Analogue Scale (VAS) over the last 7 days in PD patients after 4 sessions of FR at a rate of one hour session every 3 weeks compared to a group of patients receiving a sham massage (SM) of the same duration and frequency.    **The secondary objectives are:**   1. To compare the number of patients with at least 30% improvement in pain on VAS after management between the 2 groups of patients (FR vs SM). 2. To compare the kinetic variation in the analgesic effect of a 4-session FR treatment on the intensity of chronic pain measured by a Visual Analogue Scale (VAS) during management between the 2 groups of (FR vs SM). 3. To compare the variation in chronic pain using the King's Parkinson's disease Pain Scale (KPPS), the Brief Pain Inventory (BPI), and the Primary Parkinsonian Pain Diagnostic Questionnaire (3PDQ), before and after management between the 2 groups of patients (FR vs SM). 4. To compare the evolution of the subjective nociceptive threshold and tolerance to |

|  | hot by means of the thermotest between the 2 groups of patients (FR vs SM):   1. before and after each session of FR vs SM at V1 and V4 2. before and after overall RP or MS support between V1 and V4 3. Compare the consumption of analgesics using a record book of analgesic treatments throughout the study, between the 2 groups of patients (FR vs SM). 4. To compare pain acceptance with CPAQ-8, anxiety and depression with HAD, before and after management between the 2 groups of patients (FR vs SM). 5. To determine if there are biomarkers of the effect of RP on brain activity using fMRI before and after management between the 2 groups of patients (FR vs SM). 6. To assess whether there are personality traits that modulate the analgesic response in the 2 groups of patients (according to the results obtained in the TCI personality test) |
| --- | --- |
| **JUDGING CRITERIA** | **Primary outcome** :  The primary outcome of this study is the variation in mean pain intensity during the previous week, rated on an VAS (from 0 = no pain to 10 = maximum imaginable pain), before and after management between the 2 groups of patients (FR vs SM). Pain intensity on VAS is a validated clinical criterion for the global assessment of the discriminative and emotional aspects of pain [Price 1994].  The variation corresponds to the raw delta between the VAS score at baseline (V1) and that after the 4 sessions of FR or SM (V5). **Secondary outcomes :**  The impact of the management in the 2 groups of patients (FR vs SM) will also be evaluated through:   1. The number of responding patients, i.e. who have at least a 30% improvement in their pain on VAS after overall management. 2. To the kinetics of the pain intensity curve between the VAS score (over the last 7 days) at baseline (V1) and that at each visit. There are therefore 4 mean deltas for each group of patients (FR vs SM). 3. At the deltas of the following scores, before and after global management:    - KPPS    - of the BPI    - of the Mac Gill    - of the 3PDQ 4. At the subjective pain perception threshold at heat and heat tolerance. For each patient in ON* condition:    - before and after each session of FR or SM at V1 and V4    - before and after overall FR or SM support between V1 and V4 5. Consumption of analgesics by means of a collection booklet throughout the study (collection of the prescribed treatment and dosage) 6. At the deltas of the following scores, before and after global management:   a) acceptance of pain |

|  | b) of the HAD scale   1. Comparison between the structural and activation maps of brain networks in order to identify biomarkers of the management of brain activity. The modulation of brain activity will also be assessed by means of a map of correlation coefficients representing the strength of connectivity between the different brain networks and regions of interest, obtained from functional MRI sequences at rest, [after – before] care. 2. Comparison between the 2 groups of patients of their analgesic response according to their personality profile |
| --- | --- |
| **RESEARCH OUTLINE** | This is a single-center, longitudinal, prospective, double-blind, randomized exploratory study in 2 parallel groups (1:1 ratio): 1 group benefiting from PR sessions, 1 group benefiting from foot SM sessions. |
| **CRITERIA**  **INCLUSION** | - Men and women ≥ 18 years old. - Parkinson's disease with little fluctuation, i.e. with MDS UPDRS IV ≤ 2. - Patients who have chronic pain of any origin, regardless of the pathophysiological mechanism for at least 3 months with VAS ≥ 4. - Patients whose anti-Parkinson's treatment has been stable throughout the duration of the study and at least for 4 weeks. - Ability to provide informed consent. - Affiliation to a social security scheme or equivalent. |
| **CRITERIA FOR NON-INCLUSION** | - Patients with cognitive impairment defined by a MOCA < 25. - Patient included in a clinical trial that potentially interferes with the purpose of the study (interventional study, early study for a drug). - Patient refractory to foot massage. - Patient who has benefited from a PR session in the last 6 months. - Contraindication to foot reflexology: history of phlebitis less than 3 months old; skin lesions on the feet or unresolved fractures on the feet. - Patients with a contraindication to MRI: claustrophobic patient, patient with a metallic element. - Patient refusing to be informed of any abnormality detected on brain MRI. - Protected patient: adults under guardianship, curatorship or other legal protection, deprived of liberty by judicial or administrative decision. |
| **TREATMENTS/**  **RESEARCH STRATEGIES/ PROCEDURES** | Patients in the **experimental group** will receive 4 FR sessions, of one hour, spaced 3 weeks apart each. The FR will be carried out by Emeline Descamps, CNRS researcher, seconded to INSERM, trained and certified in reflexology. The specific PR protocol used in the present study was designed and standardized in collaboration with experienced reflexology practitioners. (It will be the same for all patients and for all sessions). This protocol stimulates the reflex zones associated with the management of pain, stress and emotions, it is described in part 7 "Treatment/Research strategies and procedures".    **Patients in the control group** will receive 4 sessions of SM, feet and calves, (without stimulating the foot reflexology points) of one hour, spaced 3 weeks apart by the same practitioner. This massage has been standardized and will be the same for all patients in this group and for all sessions. |
| **STUDY SIZE** | We will include 30 patients in this study divided into 2 groups of 15 patients. |
| **DURATION OF THE SEARCH** | Length of Inclusion Period: 2021 – 2023  Duration of participation of each participant: 12 weeks Total duration of the research: 3 years |
| **ANALYSIS**  **STATISTICS OF**  **DATA** | Descriptive, mean, standard deviation and extent or median and interquartile analysis will be presented for quantitative variables. For qualitative variables, it will be a question of number and percentage.  For comparative analyses between the 2 groups:   - The non-parametric Wilcoxon Mann-Whitney test will be performed for quantitative variables - The nonparametric Wilcoxon test for paired data will be used for before-and-after comparisons for quantitative variables - The Chi2 test (or Fisher if theoretical < 5) will be performed for the qualitative variables - The McNemar test will be used for before-and-after comparisons for qualitative variables. |
| **EXPECTED BENEFITS** | To date, it is clear that very few good quality studies have been carried out on the subject. Here we propose to scientifically and rigorously evaluate foot reflexology in order to reach reliable conclusions in pain in Parkinson's patients. The challenge is to provide concrete help to Parkinson's pain patients and to enable doctors to recommend this type of care in an informed manner. |

*in ON condition; that is to say, at least one hour after taking the anti-parkinsonian treatment for the treatment to be fully effective.

AbstractThis research has been registered in http://www.clinicaltrials.gov/.

Exploratory study of the impact of foot reflexology on chronic pain in parkinsonian patients. University Hospital Toulouse is the sponsor of this research.

**Brief summary**

Pain is one of the non-motor symptoms of Parkinson's disease still poorly known and misdiagnosed and its management is complex. This statement encourages us to explore new non-drug therapeutic paths, such as foot reflexology (FR). We propose in this study to compare the evolution of different parameters, quantitative and qualitative, and to identify biomarkers and highlight the specific effect of FR on pain, compared to sham foot massage (SM).

**Detailed description**

Parkinson's disease (PD) is a neurodegenerative disease characterized by the destruction of specific neurons involved in movement control. Pain is one of the non-motor symptoms still poorly known and misdiagnosed. In addition to this fact, its management is complex. Currently, the medical community has no effective solution to reduce pain to an acceptable level in terms of intensity or frequency.

Pain is a personal, subjective experience with an affective and cognitive dimension. The context and its psychological impact can amplify as well as alleviate the pain. Thus, non-pharmacological treatment (NPI) that influence the psychological state (mood, level of stress, ...) can also modulate the pain experience through the brain matrix of pain. Among them, foot reflexology, which by stimulating reflex zones located on the feet, makes it possible to modify the perception of the pain and to modify the subjective / emotional valence of the pain. In this study, we propose to evaluate this technique, by postulating that it should reduce the chronic pain of patients with Parkinson's disease.

This project is innovative and original in two dimensions: On the one hand, the pain of patients in PD is still often underestimated, there is in the mere fact of devoting a study a real dynamic of changes in the consideration of the patients concerned and in the management of this non-motor symptom. On the other hand, the increasing use of NPI requires that clinical studies be conducted. To date, it is clear that very few or no reliable studies have been conducted on the subject. We wish to develop complementary nonpharmacological management to provide concrete help to Parkinson's patients and thus initiate the scientific validation of NPI as recommended by the High Authority of Health.

**The Primary outcome** is to evaluate the variation in the mean intensity of chronic pain by FR measured using a Visual Analog Scale (VAS) over the last 7 days in patients with PD after 4 sessions of FR at the rate of one session 1 hour every 3 weeks compared to a group of patients receiving a sham massage (SM) of the same duration and at the same frequency.

**The Secondary outcomes** will consist of determining if there are markers of the effect of FR about: (1) the improvement in pain on the VAS, (2) the kinetic variation of the analgesic effect, (3) the variation in chronic pain using the King's Parkinson's disease Pain Scale (KPPS), Brief Pain Inventory (BPI), and Primary Parkinsonian Pain Diagnostic Questionnaire (3PDQ), (4) the evolution of the subjective nociceptive threshold and heat tolerance evaluated by the thermotest, (5) the consumption of analgesics using a logbook of analgesic treatments, (6) pain acceptance, anxiety and depression and (7) brain activity. The personnality will be evaluated by Temperament and Character Inventory (TCI) questionnaire.

**Study design**

The proposed plan to validate the hypothesis is a monocentric, longitudinal, prospective, double blind (patient and assessor), comparative, randomized exploratory study in parkinsonian patients, with minimal risk and constraints.

**Eligibility criteria**


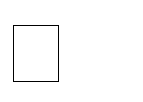
 Inclusion criteria: Adult ≥ 18, Little fluctuating Parkinson's disease attested by a score ≤ 2 on the MDS UPDRS IV, Patients with chronic pain for at least 3 months with a VAS ≥ 4 and Patients whose antiparkinsonian treatment is stable throughout the duration of the study and at least for 4 weeks.


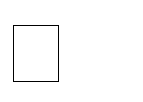
 Exclusion criteria: Patients with cognitive impairment defined by an MOCA <25. Contraindication to carrying out the MRI examination (Pregnant or lactating woman, claustrophobic patient, patient carrying a metallic element), Patient refusing to be informed of any abnormality detected on brain MRI, Patient included in a clinical trial that potentially interferes with the objective of the study, Patient resistant to foot massage, Patient having benefited from a FR session in the last 6 months, skin lesions in the feet, recent fracture in the feet, history of phlebitis less than 3 months and history of deep vein thrombosis.

Arm number or label and arm type

Sham massage (SM) comparator will be a placebo massage of FR.

Two arm will be received the FR and SM.

**Interventions**

The experimental group will receive 4 one-hour FR sessions spaced 3 weeks apart. FR will be performed by a researcher trained and certified in reflexology. The specific FR protocol used in this study was designed in collaboration with experienced reflexology practitioners and will be the same for all patients and for all sessions. This protocol stimulates the reflex zones associated with the management of pain, stress and emotions.

The control group will receive 4 sessions of SM, feet and calves, one hour, spaced 3 weeks apart, performed by the same researcher. This massage will be the same for all patients in this group and for all sessions. The estimated duration of the inclusion period is estimated to two years. The duration of each participant's participation is established to 12 weeks. The total duration of the research is established to three years.

**Number of subjects**

30 patients divided into 2 groups of 15 patients

**Statistical analysis**

A description of the characteristics of the population at inclusion will be made.

Descriptive, mean, standard deviation and range or median and interquartile analysis will be presented for the quantitative variables. For qualitative variables, it will be number and percentage.

For comparative analyzes between the 2 groups : The Wilcoxon Mann-Whitney nonparametric test, Wilcoxon's nonparametric paired test, The Chi2 test (or Fisher if theoretical numbers <5) and McNemar's test.

The fMRI images will be analyzed according to standard procedures in the literature in this field. First, we will perform signal processing and spatial image processing (slice timing, realignment, normalization, smoothing). For the fMRI approach at rest, the images will be analyzed using a model-dependent method.

**Conditions:** Parkinson disease and pain

**Key-words:** Parkinson disease, reflexology, pain, non-pharmacological treatment specific effect,.

**2. SCIENTIFIC RATIONALE AND GENERAL DESCRIPTION**

## 1. CURRENT STATE OF KNOWLEDGE

Chronic pain in Parkinson's disease (PD) is particularly common and has a major biopsychosocial impact. Its prevalence is high, estimated at between 60 and 80% on the basis of several epidemiological studies *[Beiske 2009; Defazio 2008; Nègre-Pages 2008]*. Thus, a large-scale pharmacoepidemiological study carried out on more than 11000 Parkinson's patients evaluated the chronic consumption of analgesic drugs in Parkinson's patients using a database of the Assurance Maladie in France *[Brefel-Courbon 2009].* This study showed that the chronic prescription of analgesics was higher in Parkinson's patients compared to the general population and was substantially identical to that of patients with rheumatic pathologies. Among analgesic drugs, opioid drugs and paracetamol were the most frequently consumed by Parkinson's patients compared to the general population. The same was true for certain antiepileptics and antidepressants indicated for neuropathic pain.

**However, pain is one of the non-motor symptoms that is still poorly understood and misdiagnosed in Parkinson's patients.**

The diagnosis of chronic pain in Parkinson's patients is particularly difficult because of the multiplicity of types of pain, which result in distinct mechanisms, especially since Parkinson's patients can present several types of pain at the same time *[Beiske 2009].* Some pain is linked to a dysfunction of the central pain integration system, while other pain is linked to the impact of the disease on the muscular and joint system.

There are 3 main types of pain according to the new classification of Marques et al *[Marques, 2019]:*

- **Nociceptive pain which can be:**
- **dystonias, specific to PD.**
- **musculoskeletal pain, which is not specific to PD.**
- **Neuropathic pain called radicular, not specific to PD.**
- **Harmful plastic pains which can be:**
- **central parkinsonian pain, specific to PD.**
- **restless leg syndrome, which is not specific to PD.**

**This classification shows the complexity of making a diagnosis.**

Thus, pain is still too often insufficiently taken into account and its management remains complex. At present, the medical profession does not have an effective solution to reduce it to an acceptable level in terms of intensity or frequency. No reliable study has been able to demonstrate the effectiveness of drug treatment in the management of this pain or its consequences on patients' quality of life. This encourages caregivers to explore new non-drug therapeutic avenues, complementary to conventional treatments.

Pain is above all a personal, subjective experience with an affective and cognitive dimension.

The context and its psychological impact can amplify as well as alleviate pain *[Koechlin 2018]*. Thus, non-drug interventions (NPIs) that influence psychological state (mood, stress level, etc.) can also modulate the experience of pain, particularly on these psychological and pain acceptance aspects *[Ball 2017].*

Among them, foot reflexology (RP) *[Byers 1983, 2001],* which, by stimulating reflex zones located on the feet, makes it possible to modify the subjective/emotional valence of pain and thus to modify its perception. It is described as a promising therapeutic method in the management of stress *[Hughes 2011]*, pain *[Stephenson 2003, Hughes 2009]* and in improving quality of life *[Wyatt 2012]* in the general population. Foot reflexology is a specialized massage that **involves applying controlled pressure to specific areas of the feet**, called reflexes. It is based on the premise that these reflex zones are correlated with organs, glands, or systems of the human body *[Stephenson 2007, Poole 2007].* By exerting these pressures, practitioners aim to promote homeostasis and thus stimulate the body's adaptive capacities in order to restore and maintain physiological and psychological health and well-being *[Byers 1983, 2001; Faure Alderson 2008, McVicar 2007, Özdemir 2013]*.

Its concept was introduced in the United States at the beginning of the twentieth century by Dr. William Fitzgerald, an ENT surgeon. He reported that local pressure on a finger or toe had a definite effect and brought physiological functions back to normal, regardless of the distance between the treatment area (finger or toe) and the part of the body he wanted to act. He thus presented a map called the "Ten Zone Theory" *[Fitzgerald 1917],* which shows longitudinal and vertical areas running from the top of the head to the tips of the toes. Over the years, empirically, RP boards have been enriched with more and more precise reflex zones, schematized in Figure 1, this led to the first publication in a medical journal in 1993 *[Oleson and Flocco 1993].*

**This figure has been removed due to copyright restrictions.**

Figure 1: "Diagram of the zones" Excerpt from Total Therapy Reflexology, M. Faure Alderson.

While the current state of the art of FR is not based on many large-scale trials, there is clinical **evidence of the effectiveness of RP on the physical and emotional health of those who benefit from it**. In 1990, it was introduced as supportive care in oncology in the USA *[Wilkinson 2008]* and since 2010, in France, but in an arbitrary and uncoordinated manner.

This well-tolerated MNI *[Donley 2018]* could be a complementary solution in the management of pain in Parkinson's patients, whose daily drug treatment is heavy and complex.

Our team *[Brefel-Courbon 2009]* showed that the repeated prescription of analgesics was higher in Parkinson's patients compared to the general population and was more or less identical to that of patients with rheumatic pathologies. We think that RP could also impact this consumption of analgesics.

In this study, we propose to evaluate the impact of RP on the intensity of chronic pain, postulating that it will reduce chronic pain in patients with Parkinson's disease.

## RESEARCH HYPOTHESES AND EXPECTED RESULTS

There is no research on the subject at this time.

We hypothesize that FR will have an effect on pain in patients with Parkinson's disease. Reflexology acts on physical and emotional health, we believe that management with FR could improve chronic pain, anxiety and depression and reduce the use of analgesics in this population.

## JUSTIFICATION OF METHODOLOGICAL CHOICES

Critics of non-drug interventions and FR claim that the effect is only placebo or related to management and patient/therapist interaction. This is why, in the present study, we propose to study the impact of FR management compared to a sham massage (SM), with the same reflexologist practitioner, in order to avoid a bias related to the personality of the therapist and therefore to the patient's care.

In addition, the patient will be informed that he or she will be randomly selected to benefit from an analgesic NPI, explaining that two different techniques are being studied in this protocol. The patient will thus be blinded.

In order to ensure objective evaluation and blinded data analysis, the person in charge of pain assessment of Parkinson's patients will not be informed of the patient's randomization. This is why we talk about double-blind in this study.

The rigorous evaluation of pain in this study led us to select many questionnaires that evaluate the different aspects of pain. This will allow us to cross-reference the different information in order to strengthen the observation of the impact of the management of FR.

## BENEFIT/RISK RATIO

Participants in the experimental group of the DOREPAR study will have a direct individual benefit in the short term since we postulate that the treatment will allow an improvement in chronic pain in patients with PD. Patients in the control group (SM) will benefit from close care and follow-up that includes massages.

In the long term, the benefit should be collective because it will make it possible to better manage pain, which can be particularly disabling for Parkinson's patients with an expansion of the care offer.

There is no risk associated with this research.

We therefore consider this research to have a favourable benefit/risk ratio.

## EXPECTED BENEFITS

This study proposes an **innovative approach** to the management of chronic pain for Parkinson's patients in this study by RP.

▪ **Expected outcomes directly for patients in the study**

Through this project, we propose a FR protocol whose content has been standardized, exploring the effectiveness of this management through different markers and scales, compared to an SM. This study will thus make it possible to better specify the uses and represents an essential step in validating the impact of FR on patients' chronic pain in a comparative approach. Based on experience and observation, we postulate that this management (of 4 FR sessions) should lead to clinically relevant improvement in chronic pain.

- **Preliminary data: solid basis for a larger study**

All the elements collected will constitute preliminary data that will allow us to subsequently design a larger research project. These data will make it possible to validate their effectiveness in terms of benefits for patients (reduction of chronic pain, quality of life, stress management), as well as to propose brain imaging biomarkers of potential brain changes that occur during pain reduction in PD patients.

- **Expected long-term impact on the Parkinson's patient population** In the population of Parkinson's patients with chronic pain, the use of FR could be beneficial in terms of:
- **by improving the perception of pain and therefore the daily life of patients,**
- **by avoiding the use of pharmacological analgesics and their possible side effects.**

In the long term, the challenge is to provide new tools in the management of chronic pain, to have reliable information to better inform patients and to enable health professionals to recommend RP as a complementary therapeutic method in an informed manner.

## RATIONALE FOR LOW LEVEL OF INTERVENTION

This project involves a low level of intervention since foot reflexology is a very well tolerated and non-invasive method.

The patient will have to come every 3 weeks for 3 months to benefit from either FR or SM.

Brain fMRI examination without contrast agent injection at the beginning and end of the study also presents a minimal risk.

We therefore consider that it falls within the framework of interventional research with minimal risks and constraints (category 2 of the Jardé law).

**3. RESEARCH OBJECTIVES**

## MAIN OBJECTIVE

The main objective is to evaluate the variation in the mean intensity of chronic pain by FR measured by a Visual Analogue Scale (VAS) over the last 7 days in PD patients after 4 sessions of FR at a rate of one hour session every 3 weeks compared to a group of patients receiving 4 sessions of a sham massage (SM) of the same duration and frequency.

## SECONDARY OBJECTIVES

The secondary objectives are:

1. To compare the number of patients with at least 30% improvement in pain on VAS after management between the 2 groups of patients (FR vs SM).
2. To compare the kinetic variation in the analgesic effect of a 4-session PR treatment on the intensity of chronic pain measured by a Visual Analogue Scale (VAS) during management between the 2 groups of patients (FR vs SM).
3. Comparing the variation in chronic pain using King's Parkinson's disease Pain

Scale (KPPS) and the Brief Pain Inventory (BPI), and the Primary Parkinsonian Pain Diagnostic Questionnaire (3PDQ) before and after the overall management between the 2 groups of patients (PR vs. MS).

1. Compare the evolution of the subjective nociceptive threshold and the tolerance to heat using the thermotest between the 2 groups of patients (FR vs SM):

a) before and after overall support between V1 and V4

1. Compare analgesic consumption using a diary of analgesic treatments throughout the study, before and after treatment between the 2 groups of patients (FR vs SM).
2. To compare pain acceptance with CPAQ-8, anxiety and depression with HAD, before and after management between the 2 groups of patients (PR vs. MS).
3. To determine if there are biomarkers of the effect of RP on brain activity using fMRI before and after management between the 2 groups of patients (RP vs MS).
4. To assess whether there are personality traits that modulate the analgesic response in the 2 groups of patients (according to the results obtained in the TCI personality test)

**4. JUDGING CRITERIA**

### 4. PRIMARY OUTCOME

The primary outcome of this study is the variation in mean pain intensity during the previous week, rated on an VAS (from 0 = no pain to 10 = maximum imaginable pain), before and after management between the 2 groups of patients (FR vs SM). Pain intensity on VAS is a validated clinical criterion for the global assessment of the discriminative and emotional aspects of pain *[Price 1994].*

We chose to assess the intensity of the overall pain and not the intensity of the different types of pain. Indeed, we believe that this type of intervention can have a non-specific effect on pain and therefore reduce the perception of pain in general and thus improve the daily life of patients. The variation corresponds to the raw delta between the VAS score at baseline (V1) and that after the 4 sessions of FR vs SM at the last visit (V5).

## SECONDARY OUTCOMES

The impact of the management in the 2 groups of patients (FR vs SM) will also be evaluated through:

1. The number of responding patients, i.e. who have at least a 30% improvement in their pain on VAS after overall management.
2. The kinetics of the pain intensity curve (assessed with the VAS over the last 7 days) between baseline and each visit. There are therefore 4 mean deltas for each group of patients *[Price 1994].*
3. At the deltas of the following scores, before and after global management:
   - of the KPPS *[Chaudhuri 2015]* which is a reliable and validated scale in the assessment of pain intensity in Parkinson's disease. It includes 14 questions grouped into 7 areas.

Each item is rated according to severity (between 0 and 3) and multiplied by frequency (between 0 and 4). A subscore between 0 and 12 is obtained for each item. The total score is between 0 and

168 by adding up all the sub-scores.

- - of the BPI *[Brasseur 1997]* which evaluates different dimensions of pain such as functional disability, as well as social and psychological impacts. It includes 7 items that patients rate between 0 (no discomfort) and 10 (maximum discomfort).
  - of Mac Gill *[Turk 1985]* which is a self-questionnaire that describes the type of pain and quantifies its intensity. It evaluates the sensory-discriminative dimension and the affective dimension of pain separately. There are 15 items, 8 of which are for the sensory-discriminative dimension and 7 for the affective dimension. Each item is rated between 0 (absent) and 4 (strong).
  - 3PDQ, which is a self-questionnaire currently being validated in France that would make it possible to diagnose primary Parkinson's pain. It includes 20 closed-ended questions.

1. At the threshold of subjective pain perception to heat and tolerance to heat. For each patient in ON condition (i.e. when the patient has taken his or her anti-Parkinson's treatment in the usual way) we will determine their variations:
   - - before/after the session at visit 1,
     - before/after the session at visit 4,
     - between V1 and V4 before the session is carried out,
     - between V1 and V4 after the session has been carried out, - .
     - For the threshold of subjective pain perception in warmth, thermal stimulations will be performed using a thermotest (MSA Thermotest, Somedic AB, Sweden) *[Frushstorfer 1976].* A 12x25mm contact thermode will be placed in contact with the patient's thenar eminence. We will determine the nociceptive threshold specific to each subject. To do this, we will use the threshold method which does not depend on the reaction time *[Gescheider 1985; Gracely 1987].* The determination of the pain threshold will be carried out on the hemibody most affected by the disease; This will be determined by the interview (the patient's feelings, laterality of the symptoms at the beginning of the disease) and confirmed by the clinical examination (part III of the UPDRS: rigidity, tremor and akinesia).

*Method of levels* : The initial temperature of the thermode being 30ºC, the patient will receive a temperature of +3°C for 30 seconds. Immediately after each thermal stimulation, the patient will be asked whether or not they have felt a painful sensation (yes/no); If the answer is no, the temperature will then be increased by 3ºC and applied again for 30 seconds, until the patient feels a painful sensation; In case of a positive response, the temperature will be lowered by 1.5°C (half of 3°C) and applied again for 30 seconds. The threshold will then be gradually adjusted until a temperature between two intervals close to 0.2ºC is obtained. An interval of 30 seconds was respected between each stimulation. Two sets of stimulations will be performed and the subjective threshold will be defined as the average of the two.

- - - For the tolerance to heat, starting from the subjective threshold determined above, we will determine: the Intensity-Response curve

After determining the nociceptive thresholds for heat, a series of supraliminal hot thermal stimuli will be randomly applied. Each stimulus will last 5 seconds and have a maximum intensity of 48°C and a minimum of 5°C. An interval of 60 seconds will be respected between each stimulation.

After each stimulus, the patient will be asked to rate the pain felt using a Visual Scale

Analogue (VAS from 0=no pain to 10=unbearable pain). This will allow us to establish a response intensity curve (EVA) as a function of temperature. Tolerance to heat

From the Intensity-Response curve, we will be able to determine the temperature for which the VAS was equal to 75%. This temperature will then be applied twice at five-minute intervals to the patient, who will have to orally express his tolerance threshold (which corresponds to the time before the stimulation becomes unbearable).

1. Consumption of analgesics by means of a collection booklet throughout the study (collection of the prescribed treatment and dosage).
2. At the deltas of the following scores, before and after global management:
   - CPAQ-8 pain acceptance test *[McCracken 2004]*. This self-questionnaire assesses the acceptance of chronic pain via 8 statements. Each item is rated between 0 (never true) and 6 (always true).
   - of the HAD scale *[Lépine 1985]*. It is a self-questionnaire to screen for anxiety/depressive disorders. Patients answer 14 questions: 7 about anxiety and 7 about depression. It is an assessment that measures mild to moderate depression in PPs *[Schrag 2007].*
3. Comparison between the structural and activation maps of brain networks in order to identify biomarkers of the management of brain activity. The modulation of brain activity will also be assessed by means of a map of correlation coefficients representing the strength of connectivity between the different brain networks and regions of interest, obtained from functional MRI sequences at rest, [after – before] care. The main MRI sequences performed will be resting state acquisitions (resting functional imaging) and anatomical imaging (i.e. T1 imaging).
4.
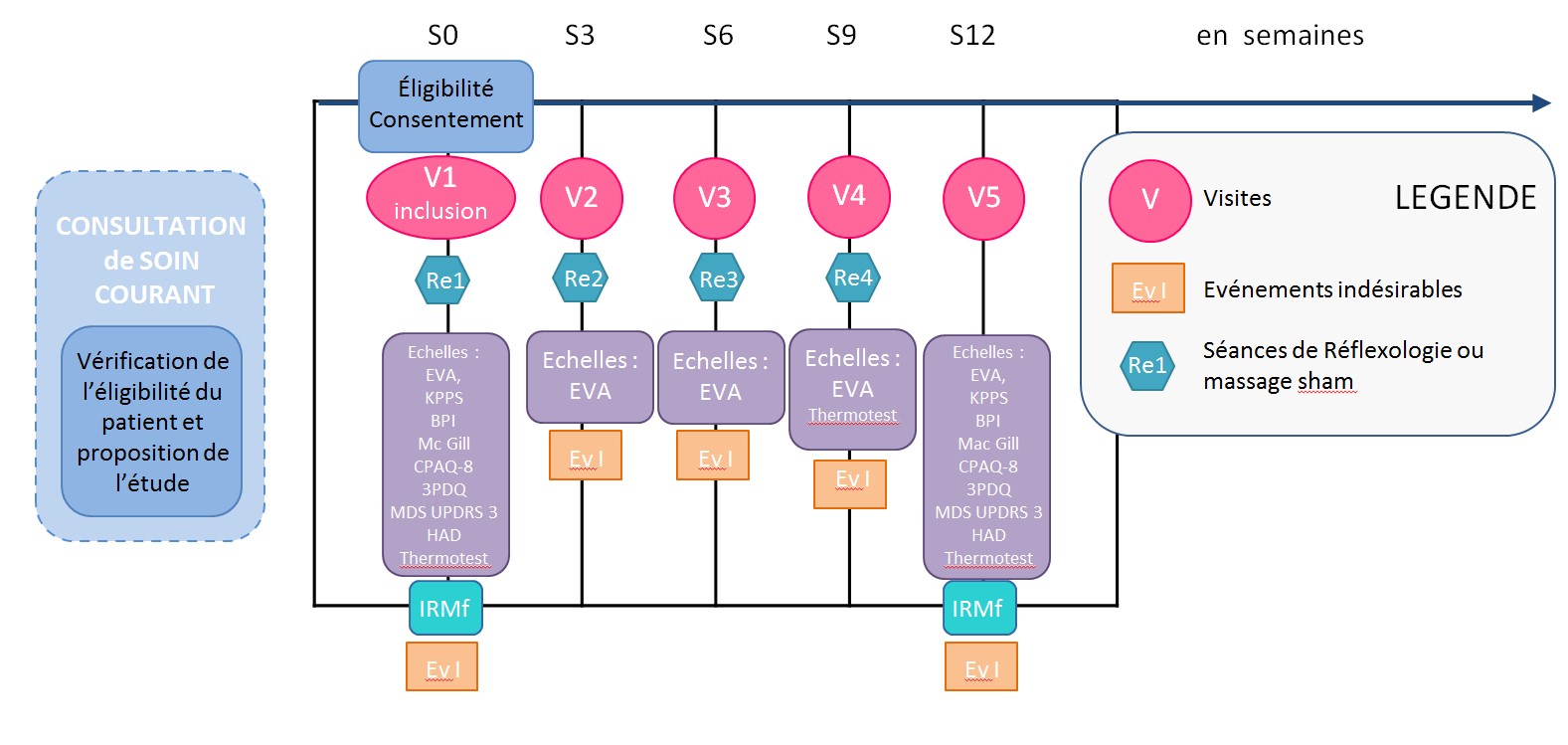
Comparison between the 2 groups of patients of their analgesic response according to their personality profile.

**5. RESEARCH DESIGN**

## RESEARCH OUTLINE

Due to the lack of published data on the subject, it was chosen to conduct a pilot study, since FR has not yet been evaluated in a population of Parkinson's patients in order to reduce their chronic pain.

It is therefore a single-center, longitudinal, prospective, double-blind, randomized exploratory study in 2 parallel groups (1:1 ratio): 1 group benefiting from FR sessions, 1 control group benefiting from foot MS sessions. Both the patient and the pain assessor will be blinded.

## METHODS FOR RANDOMIZATION

Patients will be randomized into two groups in a 1:1 ratio, the experimental group that will receive the FR sessions and the control group that will receive the foot SM.

Randomization will be established by the MeDatas cell of the Clinical Investigation Center (CIC). When a subject is included, the assignment of a number and group will be established by means of a secure randomization interface via the CIC website.

Subjects will be randomized once the investigator verifies that they meet the eligibility criteria (Section 5). Patients will be randomized to one of 2 groups according to the chronological order of inclusion of patients in the protocol.

Randomization will be done at the inclusion visit. Only the person in charge of foot reflexology and sham massages (Emeline Descamps) will be informed of the patient's randomization.

**6. ELIGIBILITY CRITERIA**

## INCLUSION CRITERIA

- Men and women ≥ 18 years old.
- Patients with no disabling dyskinesias judged by the investigator.
- Patients with chronic pain of any origin, regardless of the pathophysiological mechanism, for at least 3 months with VAS ≥ 4.
- Patients whose anti-Parkinson's treatment has been stable throughout the duration of the study and at least for 4 weeks.
- Person affiliated to or beneficiary of a social security scheme.
- Free, informed, written, and signed consent by the participant and the investigator (no later than the day of

, inclusion and prior to any review required by the research).

## CRITERIA FOR NON-INCLUSION

- Patients with cognitive impairment defined by a MOCA < 25.
- Patient included in a clinical trial that potentially interferes with the purpose of the study (interventional study, early study for a drug). - Patient refractory to foot massage.
- Patient who has benefited from a FR session in the last 6 months.
- Contraindication to foot reflexology: ATCD phlebitis less than 3 months; skin lesions on the feet or unresolved fractures on the feet.
- Patients with a contraindication to MRI: claustrophobic patient, patient with a metallic element.
- Patient refusing to be informed of any abnormality detected on brain MRI.
- Protected patient: adults under guardianship, curatorship or other legal protection, deprived of liberty by judicial or administrative decision.

## FEASIBILITY AND RECRUITMENT PROCEDURES

Patient recruitment will be carried out by the department caring for patients with PD (Toulouse University Hospital, B8 Neurology Department, Parkinson's Expert Centre (CEP)). During a routine care consultation, the neurologist will check the patient's eligibility criteria and offer to participate in the study. Patients will be re-evaluated in consultation by an investigating physician during the inclusion visit, after signing the informed consent (V1). 1500 patients are currently being monitored at the Toulouse CEP. 2/3 of Parkinson's patients suffer from pain. This active queue should allow us to recruit all patients for this study.

All the research will take place in the Baudot pavilion of the Toulouse Neuroimaging Center (ToNIC – Inserm/UPS UMR1214), which is entirely dedicated to research and located on the site of the Toulouse Purpan University Hospital. PR and MS interventions will be provided in a room dedicated to this purpose and MRIs will be performed within the MRI technical platform.

**7. RESEARCH TREATMENT(S)/STRATEGY(S)/PROCEDURE(S)**

## EXPERIMENTAL TREATMENT/STRATEGY/PROCEDURE

### *Foot reflexology*

The experimental group will receive 4 one-hour PR sessions, spaced 3 weeks apart each.

The FR will be carried out by Emeline Descamps, CNRS researcher, seconded to INSERM, trained and certified in reflexology. The specific PR protocol used in the present study has been designed in collaboration with experienced reflexology practitioners and will be identical for all patients and for all sessions. This protocol stimulates the reflex zones associated with the management of pain, stress and emotions. These sessions are already described.

A reflexology session lasts one hour and takes place in three stages:

- The pre-session dialogue: the practitioner welcomes the patient and collects some information on the patient's bodily feelings and expectations that day. (Ask the patient if there is any particular pain that needs more attention).
- Practice: The patient is comfortably seated in a massage chair, with his legs slightly raised and his feet bare. The practitioner disinfects the patient's feet and then takes them in hand, starting with a few relaxation movements to release tension and allow relaxation. A neutral massage balm will be applied and then, depending on the group, the FR or SM protocol will then be dispensed by the teacher.
- Post-session dialogue: the facilitator collects the person's sensations and feelings as well as

the reflex zones on which he dwelt, if any.

### *Description of the foot reflexology protocol*

- The reflexological massage technique is exclusively manual, it is practiced with the pulp of the thumb and index finger. The pressure of the finger is perpendicular to the surface of the skin, for a few mm, then the finger moves with a rotational pressure movement in search of the reflex zones according to the established protocol. The practitioner may perceive changes in the texture of the skin (indurated, less elastic, presence of granulations) and will emphasize these areas by various appropriate techniques adapted to the patient's feelings: pressure, tissue listening and mobilization.
- The specific reflexology protocol used in the present study was designed in collaboration with experienced reflexology practitioners (Nancy Cazaux, certified from the RTTFA school - Martine Faure-Alderson). This protocol stimulates the reflexes associated with Parkinson's disease symptomatology and emotional response:
- Starting with the left foot and then the right foot.
- Full Foot Smoothing x3
- Malleoli Smoothing x3
- Listening to the primary respiratory movement
- Circuit Spine (from the SSB to the coccyx, AR on the inner edge of the foot), starting with the left foot and then the right foot.


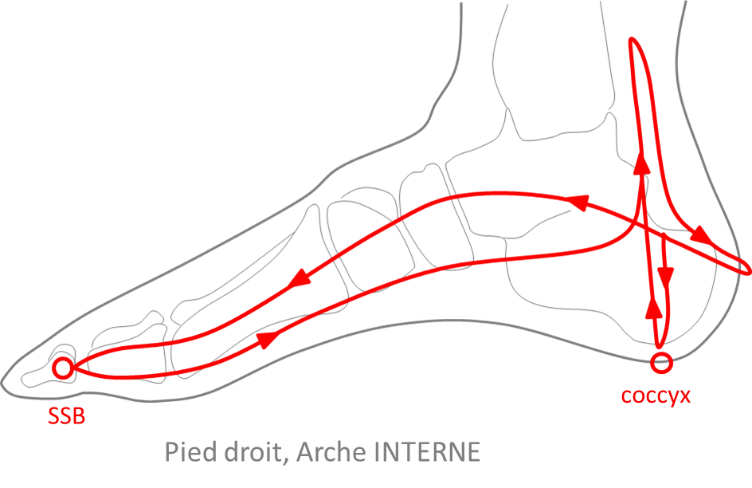


- 10 points of the brain on the big toe (Starting with the left foot and then the right foot)


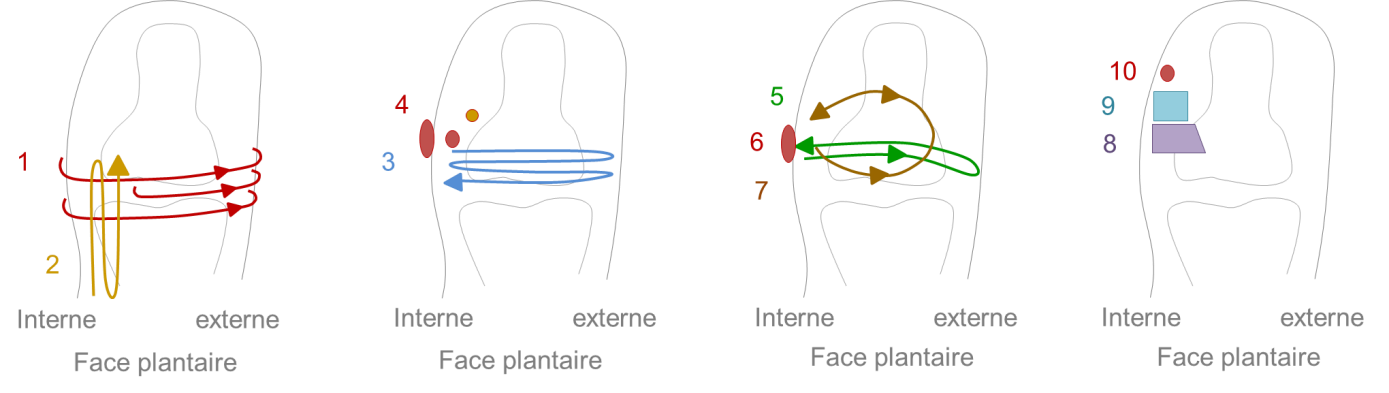


1. Occipitocervical joint
2. Brainstem, with significant stimulation on the top (locus niger) and on the middle part (raphe nuclei)
3. 4th ventricle and cerebellum
4. SSB, pituitary gland, hypothalamus (basal nucleus of meynert)
5. Cerebellum tent
6. SSB (sphenobasilar symphysis)
7. Sea horse
8. Septum pellucidum
9. Corpus callosum
10. Epiphysis

End of the Spine Circuit to the coccyx then pumping from this point (pressure / relaxation 3x)

- Circuit corresponding to emotional zones
  1. Diaphragm, 9 turns of the foot
  2. Psoas insertion, 3 foot turns

(the psoas and diaphragm insertions being intimately linked)

Insist on the stress axis: 12. adrenal glands + 13. Epiphysis, 14. Hypothalamus and Pituitary Gland: stimulate simultaneously), starting with the left foot and then the right foot.


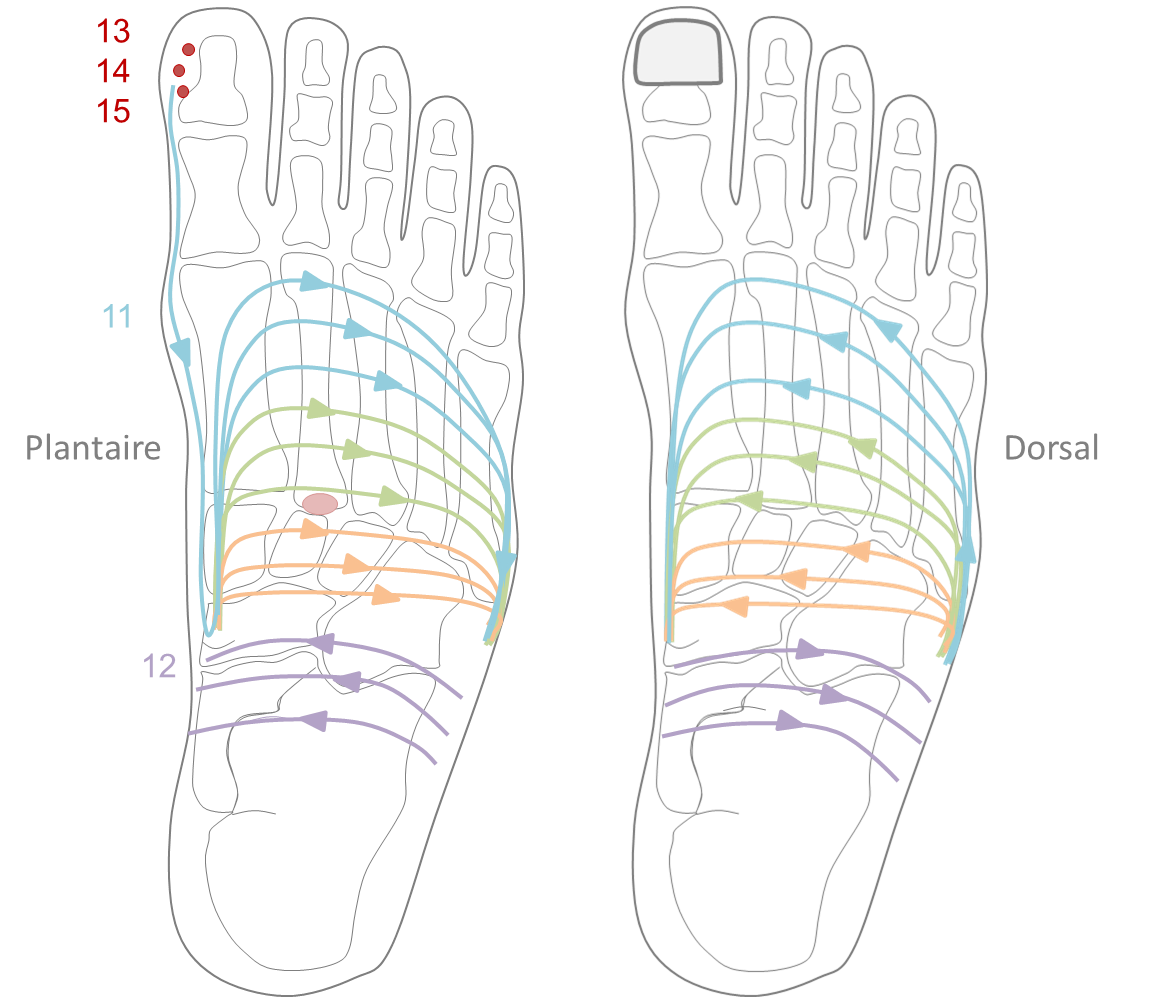


Move up and down the Spine circuit to the tailbone ▪ 7 plexiglass, starting with the left foot and then the right foot.


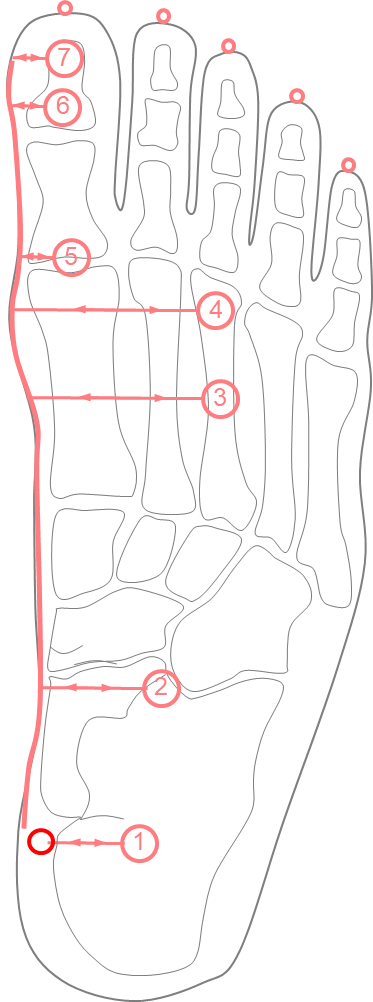


- Listening to the primary respiratory movement
- Complete smoothing of feet and malleoli

## TREATMENT/STRATEGY/COMPARISON PROCEDURE

The control group will receive 4 sessions of SM, feet and calves, of one hour, spaced 3 weeks apart by the same worker. Patients drawn at random from the group that will benefit from the SM will have a foot and calf massage without stimulating the foot reflexology points. This massage will be the same for all patients in this group and for all sessions.

The SM technique is exclusively manual, it is practiced with the hands. The pressure of the hands is perpendicular to the surface of the skin, for a few mm, and move according to the established protocol:

- Full Straightening (Foot + Calf) x3
- Malleoli Smoothing x3
- Full Calf Smoothing x3
- Inner kneading of the calf
- External kneading with the palm
- Friction
- Calf straightening x3
- Soft knee straightening
- Knee side smoothing x3
- Full Smoothing
- Heel straightening
- Pumping the hollow of the foot
- Arch pressure
- Foot smoothing
- Stress of the metatarsals
- Rotation and light toe stretching
- Foot Smoothing x3
- Smoothing between metatarsals
- Ankle Smoothing x3
- Soft-Sided Cat Paste

The content of the sessions, PR and MS, has been standardized, in order to guarantee the reproducibility of the study and its transfer to clinical practice.

**8. PROCESSING AND ASSOCIATED PROCEDURES**

## ASSOCIATED TREATMENTS/PROCEDURES ALLOWED

### Ancillary Medications

All anti-Parkinson's treatments will be taken according to the neurologist's prescription.

They will not be altered by their participation in the study.

They should be stable throughout the duration of the study and at least for 4 weeks before the initiation of the FR or SM sessions.

### Other treatments/procedures

Analgesic drugs are also authorized regardless of their pharmacological class. We will thus be able to observe the variation in the consumption of these analgesic drugs during the study. A treatment collection booklet will be provided to the patient.

## PROHIBITED ASSOCIATED TREATMENTS/PROCEDURES

All massages or reflexology sessions, outside the research program, are prohibited. During the week preceding the inclusion visit (V1) and throughout the duration of the study, all non-drug interventions are prohibited (osteopathy, acupuncture, etc.).

**9. CONDUCT OF THE RESEARCH**

## RESEARCH TIMELINE

- Length of Inclusion Period: 2 years
- Duration of participation of each participant: 12 weeks - Total duration of the research: 2 years and 3 months

## SUMMARY TABLE OF PARTICIPANT MONITORING

| 4 FR vs SM sessions every 3 weeks +/- 3 days | | | | |  | |
| --- | --- | --- | --- | --- | --- | --- |
|  | In consultation | V1 | V2 (3S) | V3 (6S) | V4 (9S) | V5 (12S) |
| Eligibility | ✓ | ✓ |  |  |  |  |
| Informed Consent (R) |  | ✓ |  |  |  |  |
| RP or MS (R) |  | ✓ | ✓ | ✓ | ✓ |  |
| EVA (S) | ✓ | ✓ | ✓ | ✓ | ✓ | ✓ |
| KPPS (R) |  | ✓ |  |  |  | ✓ |
| BPI (R) |  | ✓ |  |  |  | ✓ |
| Mac Gill (R) |  | ✓ |  |  |  | ✓ |
| CPAQ-8 (R) |  | ✓ |  |  |  | ✓ |
| 3 PDQ (R) |  | ✓ |  |  |  | ✓ |
| TCI |  | ✓ |  |  |  |  |
| Thermotest (R) |  | ✓^^[[1]](#footnote-1)^^ |  |  | ✓1 |  |
| Tolerance to Heat |  | ✓1 |  |  | ✓1 | ✓ |
| MDS UPRDS III (S) |  | ✓ |  |  |  | ✓ |
| HAD (R) |  | ✓ |  |  |  | ✓ |
| fMRI (R) |  | ✓ |  |  |  | ✓ |
| Adverse Event Collection |  | ✓ | ✓ | ✓ | ✓ | ✓ |

## PRE-INCLUSION TOUR

### Itinerary of the visit

The pre-inclusion/inclusion visit is carried out by the investigating doctor. The pre-inclusion visit takes place between 2 months and no later than 1 week before the inclusion visit. During the pre-inclusion visit, the investigating physician informs the participant and answers all questions about the objective, the nature of the constraints, the foreseeable risks and the expected benefits of the research. It also specifies the rights of the participant in the context of a search and verifies the eligibility criteria.

The pre-inclusion visit is a routine care consultation where the investigating physician verifies the patient's eligibility to participate in the study. He evaluates the patient's pain and offers him research.

A copy of the information note and consent form is then given to the participant by the investigating physician. After this information session, the participant has a period of reflection. The patient informs us of his decision by phone. If he agrees, his schedule is scheduled.

## INCLUSION VISIT / RANDOMIZATION PROCESS

### Collection of consent

Prior to any research-related review, the investigator obtains the free, informed, and written consent of the participant (or his/her legal representative if applicable).

If the participant gives his or her consent to participate, the participant and the investigator write their first and last names in plain text, date and sign the consent form. This must be signed BEFORE CARRYING OUT ANY CLINICAL OR PARACLINICAL EXAMINATION REQUIRED BY THE

RESEARCH. The consent is signed in 2 copies, one of which is delivered to him in person.

Once eligibility has been verified and consent has been signed, randomization can take place. When an investigator wishes to perform randomization after verifying the participant's eligibility, they log on to the website: http:// XX . The investigator completes the "randomization" web page after having previously confirmed all the patient's eligibility criteria on the site. After validation of the content, randomization is performed and the site immediately communicates to the investigator the unique number of the participant in the research, the result of the randomization, in particular the procedure group allocated to the patient.

Following randomization, each participant will benefit from an evaluation at inclusion:

- **Socio-demographic data (age, sex).**
- **PD treatments, analgesic treatments and concomitant treatments in progress.**
- **Pain that includes:**
  - The maximum and average VAS over the previous week *[Price 1994]*
  - The KPPS: a scale that assesses the intensity of pain in PD *[Chaudhuri 2015]*
  - The BPI *[Brasseur 1997]* - The Mac Gill *[Turk 1985]*
  - CPAQ-8: a questionnaire that assesses the extent to which the person "accepts" the presence of pain *[McCracken 2004]*
  - 3 PDQ (Parkinson's Primary Pain Questionnaire)
- **Motor state, anxiety and depression which include:** - The MDS UPRDS III scale *[Defebvre 2018]*

- HAD (Hospital Anxiety and Depression) scale *[Lépine 1985]*

- **the intensity of the pain thanks to the VAS before each FR vs SM session (from V1 to V4) over the last 7 days,**
- **an assessment of the subjective pain perception threshold and heat tolerance via the thermotest before and after the FR vs SM session**
- **An assessment of the personality profile using the TCI personality questionnaire.**

The 15 patients in each group will benefit from an fMRI before the first session of FR vs SM at the Baudot technical platform.

**Following all these evaluations, the patient will benefit from the 1st session of PR, or MS, performed by the reflexologist.**

## FOLLOW-UP VISITS

During the following visits (V2, V3 and V4), scheduled at 3-week intervals +/- 3 days. Each patient will benefit from an evaluation:

- **the intensity of the pain thanks to the VAS before each FR vs SM session over the last 7 days,**
- **a compendium of adverse events (AEs).**

**Then the patient will benefit from a FR vs SM session performed by the reflexologist.**

At visit 4, the patient will benefit from his last session of FR vs SM, the subjective nociceptive threshold and heat tolerance will be assessed before and after the session.

## END-OF-RESEARCH VISIT

The last study visit (V5) scheduled 3weeks +/- 3 days after V4 will assess all outcomes assessed in V1.

The purpose of this study is to evaluate chronic pain and not acute pain in Parkinson's patients. We will compare pain over the last 7 days of each visit throughout the study and more specifically before and after overall management with RP versus MS. The pain assessment will be carried out blinded by a clinical research associate who works in Dr. Brefel-Courbon's department and who regularly treats Parkinson's patients.

The patient will therefore not benefit from a FR vs SM session at this visit.

He will also have an evaluation of the subjective nociceptive threshold and the tolerance to heat to observe a possible residual effect of the management by foot reflexology.

At the end of this visit, the patient will have completed his participation in the study.

Patients randomized to the MS group will be able to benefit from a PR session following their participation.

The care of patients will be continued as part of the routine care for their Parkinson's disease, at the end of this visit.

There is no deferral period for this study.

## RULES FOR STOPPING A PERSON'S PARTICIPATION IN RESEARCH

The rules for stopping participation in research will be as follows

- Withdrawal of consent (voluntary decision of the participant)
- Adverse event not allowing the patient to continue
- Modification of the anti-parkinsonian treatment during the study
- Major deviation from the protocol.

In the event of termination of participation in the study, patients will continue their usual medical follow-up, without any modification. An end-of-study sheet will be completed.

There are no plans to replace patients who leave the study prematurely.

## CONSTRAINTS RELATED TO THE SEARCH AND POSSIBLE COMPENSATION OF PARTICIPANTS

The patient will be required to attend all 5 visits to benefit from the FR vs SM sessions and research-related assessments. He or she will also have to perform 2 fMRI examinations and agree to be informed if an abnormality is detected during the examination.

The patient will have an assessment of pain tolerance. This evaluation may be unpleasant but only lasts a few seconds for the patient.

Transport will be reimbursed in actual costs.

There is no provision for compensation for participants in this research.

Patients will not be registered in the national file of people who are suitable for research.

There will be no deferral period for another research at the end of this research. Patients should not participate in a clinical trial at the same time.

## 10. MANAGEMENT OF ADVERSE EVENTS / ADVERSE REACTIONS / INCIDENTS

Adverse events / adverse effects / incidents must be reported to the various health vigilance circuits applicable to each product or practice concerned (vigilance of care, pharmacovigilance, materiovigilance, haemovigilance, cosmetovigilance, etc.) in accordance with the regulations in force.

Registrants must specify that the patient is included in a clinical trial and accurately identify the clinical trial concerned.

If the investigator becomes aware of a patient safety breach in the course of the research, the investigator must promptly inform the sponsor.

**11. STATISTICAL ASPECTS**

### CALCULATION OF THE STUDY SIZE

We will include 30 patients in this study divided into 2 groups of 15 patients. The calculation is based **on the expertise of Centre Expert Parkinson** in Parkinson's disease and more particularly in the associated pain and **on data from the literature**. In assessing pain in

Parkinson's disease by means of VAS, an average reduction of 20 mm on EVA is considered to be

clinically significant (bilateral test), with an alpha risk = 0.05 and a beta risk = 0.20. The standard deviation is 1.5 on an AVE in Parkinson's patients.

With these data and by calculating the number of subjects, 12 patients per group appear to be necessary. With an estimated risk of patients leaving the study at 25%, it is therefore necessary to recruit 15 patients per group.

**THIS CALCULATION WAS CARRIED OUT BY THE RESEARCH METHODOLOGICAL SUPPORT UNIT.**

### STATISTICAL METHODS USED

Data analysis:

The data processing that will be carried out will be in accordance with the MR-001 reference methodology and therefore meets the CNIL and GDPR expectations, the data in this study will be pseudonymized and protected. A randomization list will be created to randomly determine patient groups.

A detailed flow-chart will describe the flow of subjects in the study. In particular, the number of subjects screened for eligibility, the number of eligible subjects, the number of subjects included and followed up at each visit will be mentioned. The reasons for refusal to participate and to leave the study will be described at each stage.

A descriptive analysis of the entire recruited population will be carried out to verify if there are any deviations from the protocol at the time of inclusion. Quantitative variables will be expressed as ± average standard deviation, range, medians and interquartile ranges, and qualitative variables will be expressed as numbers and percentages.

The analysis is conducted on the intention-to-treat population.

To meet the main objective, we will first describe the VAS score at Baseline (V1) and after all PR sessions (V5) by means of a "box plot" graph. This is in order to better understand the distribution of scores in the 2-stroke and in the 2 groups (as well as 4 box plots). Then we will calculate and describe the variations between V5 and V1. The variation between V1 and V5 is defined by the crude delta between these 2 visits. Finally, we will perform a non-parametric Wilcoxon Mann Whitney test to compare the distribution of variations between the 2 groups of patients.

To meet the secondary outcomes:

1. We will calculate the number of patients with a minimum of 30% improvement on the VAS score between V1 and V5. Thus, if (V5-V1)/V1 is greater than or equal to 30%, the patient is considered to be a responder. We will compare these responder rates between the 2 groups of patients using a Chi2 test.
2. We will perform a non-parametric Wilcoxon Mann Whitney test to compare the distribution of variations at each visit (delta with baseline) between the 2 groups of patients: i.e. 4 tests.
3. For the KPPS and BPI scores and the 3PDQ, we will perform the same analyses as the primary outcome because they are quantitative scores.
4. For the subjective pain perception threshold to heat and heat tolerance. We will perform the same analyses as the primary outcome as these are quantitative scores. 5. For the consumption of analgesics, which will be considered as several qualitative variables (e.g. taking paracetamol yes/no, aspirin taking yes/no), we will compare the proportion of patients taking each analgesic between the 2 groups by means of Chi2 tests.
5. The analyses regarding the CPAQ-8 score will be the same as those for the primary objective as it is a quantitative score. As the anxiety and depression scores of the HAD scale are quantitative variables, they will be analysed in the same way as the primary outcome.
6. As imaging results are quantitative variables, we will analyze them in the same way as for the primary purpose.
7. Personality profile results will be analyzed using a nonparametric Wilcoxon Mann Whitney test

For the study as a whole, the significance threshold is set at 0.05 (bilateral).

The statistical analysis will be conducted by Vanessa Rousseau from the MeDatAS – CIC – CHU Toulouse team. Analyses will be conducted using SAS analysis software version 9.4 or later.

All analyses of neuroimaging data will be carried out within the ToNIC unit (Inserm/UPS UMR1214):

- the structural and activation images obtained in MRI for each subject will be normalized and smoothed using the Statistical Parametric Mapping ((SPM) software Wellcome Department of Cognitive Neurology, University College, London, UK).
- The resting state images fMRI will be analyzed using a model-dependent method called "seed-tovoxels" using the Conn software version 18b (https://www.nitrc.org/projects/conn). The seeds will be determined according to anatomical atlases to identify the brain areas of interest (default mode network, sensorimotor network). The connectivity between these seeds and the rest of the brain voxels will be explored.

These images will then be used to perform a t-corrected test for multiple comparisons to compare the cards [after – before] support. Thus, the intensity and size of the synchronous activity of brain areas functionally connected to the areas of interest of the reflex zones stimulated on the foot can be determined. Areas of interest are defined anatomical areas in known and classic stereotactic spaces in neuroimaging.

#### 12. OVERSIGHT OF RESEARCH

RP is a very well-tolerated non-drug intervention. There will be no oversight committee for this study.

**13. RIGHTS OF ACCESS TO SOURCE DATA AND DOCUMENTS**

### ACCESS TO DATA

Acceptance of participation in the protocol implies that the investigators will make available the documents and individual data strictly necessary for the monitoring, quality control and audit of the research, available to persons with access to these documents in accordance with the legislative and regulatory provisions in force.

### SOURCE DATA

All information contained in original documents, or in authenticated copies of such documents, relating to clinical examinations, observations or other activities carried out in the context of research and necessary for the reconstruction and evaluation of the research. The documents in which the source data is saved are called the source documents. All rating scales will be entered directly into the CRF.

### DATA PRIVACY

In accordance with the legislative provisions in force, persons with direct access to the source data will take all necessary precautions to ensure the confidentiality of information relating to investigational medicinal products, research, persons who are eligible for it, and in particular with regard to their identity and the results obtained. These people, like the investigators themselves, are subject to professional secrecy.

During the research or at the end of the research, the data collected on suitable persons and transmitted to the sponsor by the investigators (or any other specialized stakeholders) will be anonymized. They must not under any circumstances display the names of the persons concerned or their addresses in plain text.

The methods of coding the participants will be: the first letter of the subject's first and last name will be recorded, accompanied by a coded number specific to the research indicating the number of the group and the order of inclusion of the subjects by group.

The sponsor will ensure that each person who participates in the research has given his or her written consent for access to the individual data concerning him or her that is strictly necessary for the quality control of the research.

**14. QUALITY CONTROL AND ASSURANCE**

### DATA COLLECTION GUIDELINES

All the information required by the protocol must be recorded in the case report forms and an explanation must be provided for each missing data. Data must be collected as they are obtained, and transcribed into these notebooks in a clear and legible manner. The data will be collected on a paper case report.

### QUALITY CONTROL

A clinical research associate mandated by the sponsor visits each investigator center on a regular basis, during the implementation of the research, one or more times during the research depending on the rhythm of inclusions and at the end of the research. During these visits, and in accordance with the risk-based monitoring plan (participant, logistics, impact, resources), the following elements will be reviewed: ▪ informed consent,

- compliance with the research protocol and the procedures defined therein,
- Quality of data collected in the case report form: accuracy, missing data, consistency of data with source documents (medical records, appointment books, original lab results, etc,...), ▪ management of potential products.

All visits will be the subject of a monitoring report by written report.

### DATA MANAGEMENT

The information will be collected for each subject on a paper report book filled in by the investigators and members of the research team. This notebook includes the identification of the subject (pseudonymized code) and the follow-up data. A source document (file created for the purposes of the study) will be kept for each subject participating in the study; The observation and medical follow-up concerning the study will be recorded in this document. This source document will be retained by the principal investigator.

All brain imaging data from the study are stored on computer media by the investigating physicians or their delegates and analyzed under the responsibility of E. Descamps (ToNIC).

The observation notebook of each patient will be kept under the control of the principal investigator in the neurology department of the University Hospital, and will be reviewed by the ARC monitoring on site, before being sent to the member of the team responsible for the entry (ToNIC). Data entry will be supervised by the study's scientific leader. The terms and conditions of entry will be defined jointly with the Project Manager. Data entry and data analysis will be carried out by the MeDatAS – CIC – CHU Toulouse unit for the statistical part and by ToNIC for the MRI part.

The data will be validated in accordance with the data management plan defined jointly by the principal investigator, the data manager and the statistician of the study.

The data freeze will be performed once all data has been collected and entered and all queries have been resolved.

The unblinding can then be carried out.

### AUDIT AND INSPECTION

An audit may be carried out at any time by persons authorised by the [sponsor and](http://www.chusa.jussieu.fr/urcest/sous_cadre.php?fich=Lexique/new_index.php?isphp=0&fich=EC/legislation/DispositionslegislativesPromoteur.htm)independent of the persons conducting the research. Its objective is to verify the safety of participants and the respect of their rights, compliance with applicable regulations and the reliability of the data

An inspection may also be carried out by a competent authority (ANSM for France or EMA in the context of a European trial for example).

Auditing, as well as inspection, may be applied to all stages of research, from the development of the protocol to the publication of the results and the classification of the data used or produced in the research.

Investigators agree to comply with the sponsor's requirements for an audit and the competent authority for a research inspection.

#### 15. ETHICAL AND REGULATORY CONSIDERATIONS

The sponsor and the investigator(s) undertake to ensure that this research is carried out in accordance with Law No. 2012-300 of 5 March 2012 on research involving human beings, as well as in accordance with Good Clinical Practice (I.C.H. version 4 of 9 November 2016 and decision of 24 November 2006) and the Declaration of Helsinki (which can be found in its full version on the [http://www.wma.net website](http://www.wma.net/)).

The research shall be conducted in accordance with this protocol. Except in emergency situations requiring the implementation of specific therapeutic acts, the investigator(s) undertake to respect the protocol in all respects, in particular with regard to the collection of consent and the notification and follow-up of serious adverse events.

This search has received the favourable opinion of the Committee for the Protection of Persons (CPP) on behalf *of the CPP* (category 2).

The Toulouse University Hospital, the promoter of this research, has taken out a civil liability insurance policy with Lloyd's BARCET 19001 in accordance with the provisions of the Public Health Code.

The data recorded during this research are subject to computerized processing at Equipe MéDatAS - CIC (for the statistical part) and at ToNIC (for the IRM part) in compliance with Law No. 7817 of 6 January 1978 relating to information technology, files and freedoms amended by Law 2004-801 of 6 August 2004.

This research falls within the framework of the "Reference Methodology" (MR-001) in application of the provisions of Article 54 paragraph 5 of the amended Law of 6 January 1978 relating to information, files and freedoms. This change was approved by decision of January 5, 2006, updated on July 21, 2016. The Toulouse University Hospital has signed a commitment to comply with this "Reference Methodology".

This search is recorded on the http://clinicaltrials.gov/ website[.](http://clinicaltrials.gov/)

CHANGES TO THE PROTOCOL

Any substantial modification, i.e. any modification likely to have a significant impact on the protection of persons, on the conditions of validity and on the results of research, on the quality and safety of the products tested, on the interpretation of the scientific documents that support the conduct of the research or on the methods of conducting it, is the subject of a written amendment which is submitted to the promoter; the latter must obtain, prior to its implementation, a favourable opinion from the CPP and, where applicable, an authorisation from the ANSM.

Non-substantial changes, i.e. those that do not have a significant impact on any aspect of the research, are communicated to the PPC for information.

All modifications are validated by the sponsor, and by all the research stakeholders concerned by the modification, before submission to the CPP and, if applicable, to the ANSM. This validation may require the meeting of any committee established for the research.

All changes to the protocol should be made known to all investigators involved in the research. The investigators undertake to respect the content.

Any change that modifies the coverage of participants or the benefits, risks and constraints of the research is the subject of a new information note and a new consent form, the collection of which follows the same procedure as that mentioned above.

#### 16. RETENTION OF RESEARCH DOCUMENTS AND DATA

The following documents related to this research are archived by the investigator in accordance with Good Clinical Practice:

- ***for a period of 15 years following the end of the research*** *(research on medicinal products, medical devices or in vitro diagnostic medical devices or research not relating to a product mentioned in Article L.5311-1 of the Public Health Code),*
- The protocol and any changes to the protocol
- Case report books (copies)
- Source records of participants who have signed a consent
- All other documents and correspondence relating to the research
- Original copy of participants' signed informed consents

All these documents are under the responsibility of the investigator during the regulatory archiving period.

No displacement or destruction may be carried out without the agreement of the promoter. At the end of the regulatory archiving period, the promoter will be consulted for destruction. All data, documents and reports may be subject to audit or inspection.

## FINAL REPORT

Within one year of the end of the research or its interruption, a final report will be drawn up and signed by the sponsor and the investigator. This report will be made available to the competent authority. The sponsor shall transmit the results of the research to the CPP and, where applicable, to the ANSM in the form of a summary of the final report within one year after the end of the research.

**17. PUBLICATION RULES**

### SCIENTIFIC COMMUNICATIONS

The analysis of the data provided by the investigator centers is carried out by the MéDatAS - CIC team for the statistics and by ToNIC for the MRI data. This analysis gives rise to a written report which is submitted to the promoter, which will be forwarded to the Committee for the Protection of Persons and the competent authority.

Any written or oral communication of the results of the research must receive the prior approval of the coordinating investigator and, where applicable, of any committee set up for the research.

The coordinating/principal investigator is committed to making the results of the research available to the public, both negative and inconclusive as well as positive.

The publication of the main results mentions the name of the sponsor, all the investigators who included or followed participants in the research, the methodologists, biostatisticians and data managers who participated in the research, the vigilantes who participated in the analysis of the safety of the participants, the members of the committee(s) set up for the research and the possible participation of the France Parkinson association (funder). International rules for writing and publishing (ICMJE'*s The Uniform Requirements for Manuscripts*, April 2010) will be taken into account.

### COMMUNICATION OF RESULTS TO PARTICIPANTS

In accordance with Law No. 2002-303 of 4 March 2002, participants are informed, at their request, of the overall results of the research.

### TRANSFER OF DATA

Data management is provided by the University Hospital. The conditions for the transfer of all or part of the research database are decided by the research sponsor and are the subject of a written contract.

## REFERENCES

[Ball 2017] Ball E., Sharizan E., Franklin G., Rogozin ́ s E., Does mindfulness meditation improve chronic pain: A systematic review, Wolters Kluwer Health 2017, Vol 29, (00).

[Beiske 2009] Beiske A.G., et al., 2009. Pain in Parkinson's disease: Prevalence and characteristics. Bread.

141, 173-7.

[Brasseur 1997] Brasseur L. Pain treatment. Paris: Doin Publishers; 1997.

[Brefel-Courbon 2009] Brefel-Courbon C; et al, Comparison of chronic analgesic drugs prevalence in Parkinson's disease, other chronic diseases and the general population. Bread. 141, 14-8.

[Byers 1983] Byers, D.C. (1983). Better health with foot reflexology: The Ingham method of reflexology. Saint Petersburg, FL: Ingham.

[Byers 2001] Byers, D.C. (2001). Better health with foot reflexology: The Ingham method of reflexology (Rev. ed.). Saint Petersburg, FL: Ingham.

[Chaudhuri 2015]: Chaudhuri KR, King's Parkinson's disease pain scale, the first scale for pain in PD: An international validation. Mov Disord. 2015 Oct; 30(12) :1623-31.

[Defazio 2008]

[Defebvre 2018]: L. Defebvre, The MDS-UPDRS Movement Disorder Society – Unified Parkinson's Disease Rating Scale – MDS-UPDRS, Neurological Practice – CME, 9, 3, 2018, 192-194.

[Donley 2018]: Donley S., McGregor S., Wielinski C.,Nance M., Use and perceived effectiveness of complementary therapies in Parkinson's disease, Parkinsonism & Related Disorders 2019, Vol 58, p 46-49.

[Faure-Alderson 2008]: Faure-Alderson, M. 2008. "Total Reflexology: The reflex points for physical, emotional, and psychological healing" - Martine Faure-Alderson, D.O

[Fitzgerald 1917]: Fitzgerald, Wm. H. "Zone therapy; or, Relieving pain at home" Columbus, O. : I. W. Long 1917, cdl; Americana.

[Frushstorfer 1976] Method for quantitative estimation of thermal thresholds in patients. Fruhstorfer H,

Lindblom U, Schmidt WC. J Neurol Neurosurg Psychiatry. 1976 Nov; 39(11):1071-5. doi: 10.1136/jnnp.39.11.1071

[Gescheider 1985] A Gescheider, B F Sklar, C L Van Doren, R T Verrillo, "Vibrotactile forward masking: psychophysical evidence for a triplex theory of cutaneous mechanoreception" J Acoust Soc Am, 1985 Aug; 78(2):534-43.

[Gracely 1987] Reliability and validity of verbal descriptor scales of painfulness

R H Gracely, R Dubner

[HAS 2011]: HAS, Development of the Prescription of Validated Non-Drug Therapeutics Synthesis - April 2011

[Hughes 2009]: Hughes CM, Smyth S, Lowe-Strong AS, "Reflexology for the treatment of pain in people with multiple sclerosis: a double-blind randomised sham-controlled clinical trial", Mult. Scler. J. 15 (11) (2009) 1329e1338

[Hughes 2011]: Hughes, C. M., S. Krirsnakriengkrai, S. Kumar, and S. M. McDonough. 2011. "The Effect of Reflexology on the Autonomic Nervous System in Healthy Adults: A Feasibility Study. " Alternative Therapies in Health and Medicine 17 (3): 32–37.

[Jenkinson 1998] Jenkinson C, Fitzpatrick R, Peto V. "The Parkinson's disease questionnaire". Health Services Research Unit, Department of Public Health, University of Oxford, 1998.

[Johns 2010]: Johns C, Blake D, Sinclair A, "Can reflexology maintain or improve the well-being of people with Parkinson's Disease?", Complementary Therapies in Clinical Practice, 2010 vol: 16 (2) pp: 96–100 [Koechlin 2018] Koechlin H., Coakley R., Schechter N., Werner C., Kossowsky J., "The role of emotion regulation in chronic pain: A systematic literature review", Journal of Psychosomatic Research 107 (2018) 38–45.

[Lépine 1985]: Lépine J P, Godchau M, Brun P, Lempérière TH. "Evaluation of anxiety and depression in patients hospitalized in an internal medicine department". Ann Méd Psychol 1985 ; 143 : 175-89.

[Marques 2019]. How to diagnose parkinsonian central pain? Marques A, Attal N, Bouhassira D, Moisset X, Cantagrel N, Rascol O, Durif F, Brefel-Courbon C. Parkinsonism Relat Disord. 2019 Jul;

[McCracken 2004]: McCracken LM, Vowles KE, Eccleston C. "Acceptance of chronic pain : component analysis and a revised assessment method". Pain 2004; 107(1-2) :159-66.

[McCullough 2014]: Mccullough J, Liddle S, Sinclair M, Close C, Hughes C, "The Physiological and Biochemical Outcomes Associated with a Reflexology Treatment: A Systematic Review", Evid Based Complement Alternat Med., 2014, 502123.

[McVicar 2007] McVicar, A.J., Greenwood, C.R., Fewell, F., D'arcy, V., Alldridge, L.C., "Evaluation of anxiety, salivary cortisol and melatonin secretion following reflexology treatment: a pilot study in healthy individuals" Complementary Therapies in Clinical Practice 2007,13(3), 137-145.

[Miller 2012] Miller L, McIntee E., Mattison P., "Evaluation of the effetcs of reflexology on quality of life and symptomatic relief in multiple sclerosis patients with moderate to severe disability; a pilot study", Clinical Rehabilitation 2013, 27 (7) 591-598.

[Nègre-Pagès 2008]: Nègre-Pagès L, Regragui W, DoPaMip Study Group, Chronic Pain in Parkinson Disease: The Cross-sectional French DoPaMip survey, Mov Disord, 2008.

[Oleson and Flocco 1993] Oleson, T., and W. Flocco. "Randomized Controlled Study of Premenstrual Symptoms Treated with Ear, Hand, and Foot Reflexology" Obs and Gyn 1993, 82 (6): 906–11.

[Özdemir 2013] Özdemir, G., Ovayolu, N., Ovayolu, Ö., "The effect of reflexology applied on haemodialysis patients with fatigue, pain and cramps" Int J of Nursing Practice 2013, 19(3), 265-273. [Poole 2007] Poole, H., Glenn, S., Murphy, P., "A randomised controlled study of reflexology for the management of chronic low back pain". European Journal of Pain 2007, 11(8), 878-887.

[Price 1994] Price DD, Bush FM, Long S, Harkins SW. "A comparison of pain measurement characteristics of mechanical, visual, analogue and simple numerical rating scales". Pain, 1994; 56 : 217-26.25.

[Schrag 2007] Schrag A, "Depression rating scales in Parkinson's disease: critique and recommendations". Mov Disord. 2007 Jun 15; 22(8):1077-92. DOI: 10.1002/MDS.21333

[Stephenson 2003]: Stephenson, Nancy L. N., and Jo Ann Dalton. 2003. "Using Reflexology for Pain Management : A Review. " Journal of Holistic Nursing 21 (2): 179–91

[Stephenson 2007] Stephenson, Nancy L. N., Melvin Swanson, Joann Dalton, Frances J. Keefe, and Martha Engelke. 2007. "Partner-Delivered Reflexology: Effects on Cancer Pain and Anxiety" Oncology Nursing Forum 34 (1): 127–32.

[Turk 1985] Turk D C, Rudy TE, Salovey P. "The McGill Pain Questionnaire reconsidered: confirming the factor structure and examing appropriate uses". Bread 1985; 21 : 385-97.

[Wyatt 2012] Wyatt G, Sikorskii A, Rahbar MH, Victorson D, You M, "Health related quality-of-life outcomes: a reflexology trial with patients with advanced-stage breast cancer", Oncol. Nurs. Forum 39 (6) (2012) 568e577.

## ANNEXES

**Chronic Analogue Visual Scale (EVA)**

Ask the patient to rate the estimated average chronic pain in the past 7 days on the visual analogue scale:

No pain Maximum pain imaginable

0 10

**MEDIUM EVA** = |__|__|__| / 100 mm

### Concise Pain Questionnaire (BPI)

**Circle the number that best describes how in the last 24 hours, the pain has bothered your:**

|  | **A. General activity** |  |  |  |  |  |  |
| --- | --- | --- | --- | --- | --- | --- | --- |
| □ 0 | □ 1 □ 2 □ 3 □ 4 | □ 5 | □ 6 | □ 7 | □ 8 | □ 9 | □ 10 |
| Does not get in the way | **B. Mood** |  |  |  |  |  | Completely annoying |
| □ 0 | □ 1 □ 2 □ 3 □ 4 | □ 5 | □ 6 | □ 7 | □ 8 | □ 9 | □ 10 |
| Does not get in the way | **C. Ability to walk** |  |  |  |  |  | Completely annoying |
| □ 0 | □ 1 □ 2 □ 3 □ 4 | □ 5 | □ 6 | □ 7 | □ 8 | □ 9 | □ 10 |
| Does not get in the way |  |  |  |  |  |  | Completely annoying |
|  | **D. Usual work (including outside the home and domestic work)** | | | | | | |
|  |  |  |  |  |  |  |  |
| □ 0 | □ 1 □ 2 □ 3 □ 4 | □ 5 | □ 6 | □ 7 | □ 8 | □ 9 | □ 10 |
| Does not get in the way | **E. Relationships with others** |  |  |  |  |  | Completely annoying |
| □ 0 | □ 1 □ 2 □ 3 □ 4 | □ 5 | □ 6 | □ 7 | □ 8 | □ 9 | □ 10 |
| Does not get in the way | **F. Sleep** |  |  |  |  |  | Completely annoying |
| □ 0 | □ 1 □ 2 □ 3 □ 4 | □ 5 | □ 6 | □ 7 | □ 8 | □ 9 | □ 10 |
| Does not get in the way | **G. Taste for life** |  |  |  |  |  | Completely annoying |
| □ 0 | □ 1 □ 2 □ 3 □ 4 | □ 5 | □ 6 | □ 7 | □ 8 | □ 9 | □ 10 |
| Does not get in the way |  |  |  |  |  |  | Completely annoying |

**SCORE (sum of items A to G) = ____________**

**McGILL PAIN QUESTIONNAIRE – SHORT FRENCH VERSION**

1. **Please describe the type of pain you have had in the past week (check (X) one box per line)**

|  |  | **No pain** | **Light** | **Moderate** | **Strong** |
| --- | --- | --- | --- | --- | --- |
| 1 | Pulsatile | 1) | 2) | 3) | 4) |
| 2 | In flashes | 1) | 2) | 3) | 4) |
| 3 | Stabbing | 1) | 2) | 3) | 4) |
| 4 | Live | 1) | 2) | 3) | 4) |
| 5 | A cramp type | 1) | 2) | 3) | 4) |
| 6 | Gnawing | 1) | 2) | 3) | 4) |
| 7 | Hot/Burning | 1) | 2) | 3) | 4) |
| 8 | Deaf | 1) | 2) | 3) | 4) |
| 9 | Heavy | 1) | 2) | 3) | 4) |
| 10 | Sensitive to contact | 1) | 2) | 3) | 4) |
| 11 | Heartbreaking | 1) | 2) | 3) | 4) |
| 12 | Tiring – exhausting | 1) | 2) | 3) | 4) |
| 13 | To make you sick | 1) | 2) | 3) | 4) |
| 14 | Agonizing | 1) | 2) | 3) | 4) |
| 15 | Cruel – who punishes | 1) | 2) | 3) | 4) |

1. **Current Pain Intensity**

1. **No pain**
2. **Light**
3. **Moderate**
4. **Strong**
5. **Very strong**
6. **Unbearable**

**McGILL QUESTIONNAIRE SCORES :**

**SUBSCORES:**

- **Sensory-discriminative subscore (sum of items 1 to 10) = _______/40**

- **Affective subscore (sum of items 11 to 15) = _______/20**

**TOTAL SCORE (sum of the two subscores) = _______/60**

**KING'S PD PAIN SCALE (KPPS)**

| This scale is designed to define and accurately describe the different types and the pattern of pain that your patient may have experienced during the last month due to his/her Parkinson's disease or related medication.    Each symptom should be scored with respect to    **Severity:** 0 = None,   1. = Mild (symptoms present but causes little distress or disturbance to patient), 2. = Moderate (some distress or disturbance to patient), 3. = Severe (major source of distress or disturbance to patient).     **Frequency** 0 = Never   1. = Rarely (< 1/wk), 2. = Often (1/wk), 3. = Frequent (several times per week), 4. = Very Frequent (daily or all the time). |
| --- |

| **Domain 1: Musculoskeletal Pain** | **Severity**  (0 – 3) | **Frequency**  (0 – 4) | **Frequency x Severity** |
| --- | --- | --- | --- |

|  |
| --- |

1. Does the patient experience pain around their Joints?

(including arthritic pain)

|  |
| --- |

|  | **SCORE** |
| --- | --- |
|  |  |
| **Domain 2: Chronic Pain** |  |

**Domain 1 TOTAL**

|  |
| --- |

1. Does the patient experience pain deep within

the body? (A generalized constant, dull, aching pain – *central pain*)

|  |
| --- |

1. Does the patient experience pain related to an

internal organ? (For example, pain around the liver, stomach or bowel – visceral pain)

|  |
| --- |

|  | **SCORE** |
| --- | --- |
|  |  |
| **Domain 3: Fluctuation-related Pain** |  |
|  |  |

**Domain 2 TOTAL**

|  |
| --- |

1. Does the patient experience dyskinetic pain?

(pain related to abnormal involuntary movements)

|  |
| --- |

1. Does the patient experience "off" period dystonia

in a specific region? (in the area of dystonia)

|  |
| --- |

1. Does the patient experience generalized "off"

period pain? (pain in the whole body or areas distant to dystonia)

|  |
| --- |

|  | **SCORE** |  |  |
| --- | --- | --- | --- |
|  |  |  |  |
|  |  |  |  |
|  |  |  |  |
| **Domain 4: Nocturnal Pain** | **Severity**  (0 – 3) | **Frequency**  (0 – 4) | **Frequency x Severity** |

**Domain 3 TOTAL**

|  |
| --- |

1. Does the patient experience pain related to

jerking leg movements during the night (PLM) or

an unpleasant burning sensation in the legs which

improves with movement (RLS)

|  |
| --- |

1. Does the patient experience pain related to

difficulty turning in bed at night?

|  |
| --- |

|  | **SCORE** |
| --- | --- |
|  |  |
| **Domain 5: Oro-Facial Pain** |  |
|  |  |

**Domain 4 TOTAL**

| \|  \| \| --- \| | \|  \| \| --- \| | \|  \| \| --- \| |
| --- | --- | --- | --- | --- | --- |

1. Does the patient experience pain when

Chewing?

| \|  \| \| --- \| | \|  \| \| --- \| | \|  \| \| --- \| |
| --- | --- | --- | --- | --- | --- |

1. Does the patient have pain due to grinding their

teeth during the night?

| \|  \| \| --- \| | \|  \| \| --- \| | \|  \| \| --- \| |
| --- | --- | --- | --- | --- | --- |

1. Does the patient have burning mouth

syndrome?

|  |
| --- |

|  | **SCORE** |
| --- | --- |
|  |  |
| **Domain 6: Discolouration; Oedema/swelling** |  |
|  |  |

**Domain 5 TOTAL**

| \|  \| \| --- \| |  |
| --- | --- | --- |

1. Does the patient experience a burning pain in

| \| their limbs (often associated with swelling or dopaminergic treatment) \|  \|  \|  \| \| --- \| --- \| --- \| --- \| \|  \|  \|  \|  \| \| 13. Does the patients experience generalised lower abdominal pain? \| \|  \| \| --- \| \|  \| \|  \| \| --- \| \| \|  \|  \|  \|  \| \|  \| **Domain** \| **6** \| **TOTAL** \| \|  \| **SCORE** \|  \|  \| \|  \|  \|  \|  \| \| **Domain 7: Radicular Pain** \|  \|  \|  \| \|  \|  \|  \|  \| \| 14. Does the patient experience a shooting pain/ pins and needles down the limbs? \| \|  \| \| --- \| \|  \| \|  \| \| --- \| \| \|  \|  \|  \|  \| \|  \| **Domain** \| **7** \| **TOTAL** \| \|  \| **SCORE** \|  \|  \| \|  \|  \|  \|  \| \|  \|  \|  \|  \| \| **TOTAL SCORE (all domains)** \| \| \| \| |  |
| --- | --- | --- | --- | --- | --- | --- | --- | --- | --- | --- | --- | --- | --- | --- | --- | --- | --- | --- | --- | --- | --- | --- | --- | --- | --- | --- | --- | --- | --- | --- | --- | --- | --- | --- | --- | --- | --- | --- | --- | --- | --- | --- | --- | --- | --- | --- | --- | --- | --- | --- | --- | --- | --- | --- | --- | --- | --- | --- | --- | --- | --- | --- | --- | --- | --- | --- | --- | --- | --- |
|  | \|  \| \| --- \| |
|  |  |
|  | \|  \| \| --- \| |
|  | \|  \| \| --- \| |

**Comments:**

### CPAQ 8


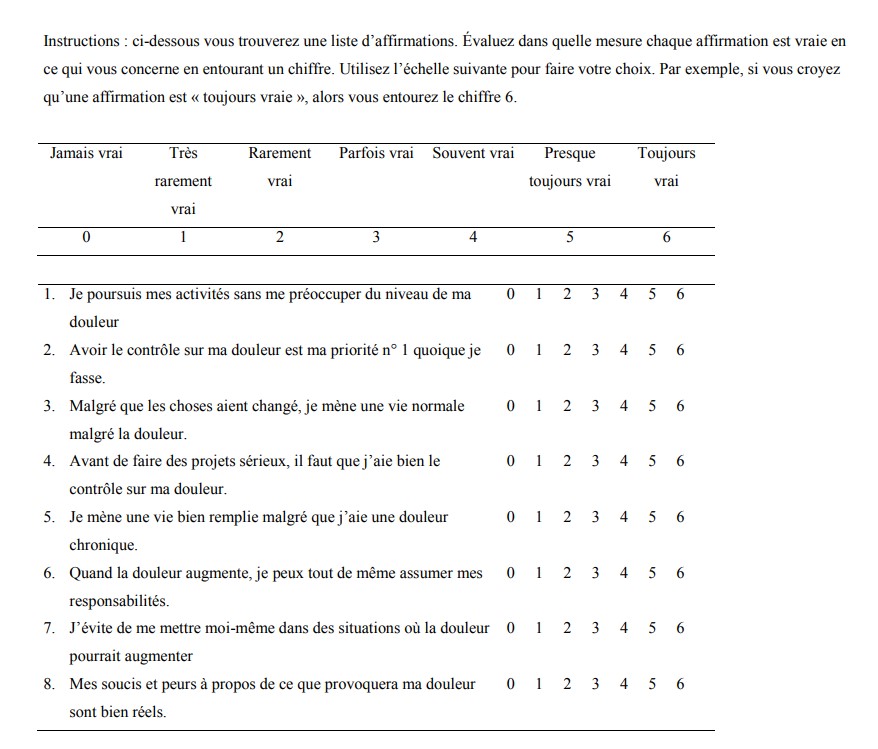


**3PDQ:**

**Parkinson's Primary Pain Questionnaire**

**This questionnaire is about your pain. If you have several pains, you should only evaluate the pain that is the most bothersome.**

- **Location of the most bothersome pain:**


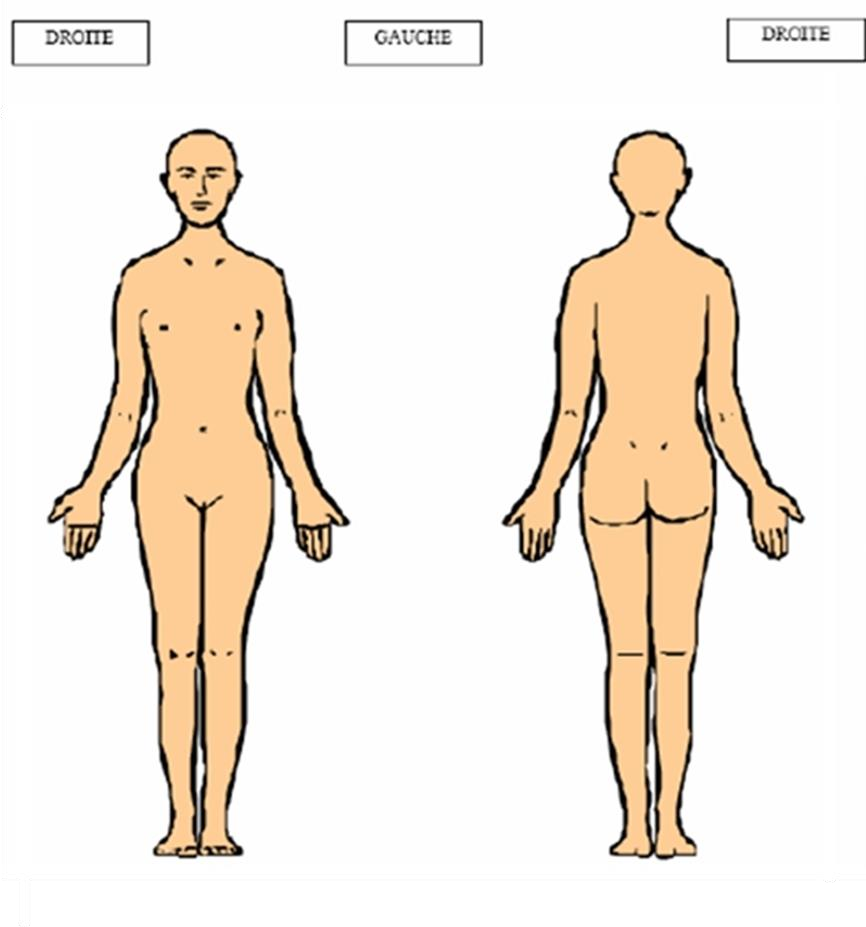


**The following questions are only about your most bothersome pain.**

- **In general, does the pain have one or more of the following characteristics:**

|  | **YES** | **NO** |
| --- | --- | --- |
| **Burn** |  |  |
| **Vice** |  |  |
| **Compression** |  |  |
| **Electric shocks** |  |  |
| **Slenderness** |  |  |
| **Painful cold** |  |  |
| **Cramp** |  |  |
| **Dull ache** |  |  |
| **Stabbing** |  |  |
| **Sting** |  |  |
| **Grinding** |  |  |
| **Deep** |  |  |
| **Nagging** |  |  |

- **In general, is the pain associated in the same area with one or more of the following symptoms:**

|  | **YES** | **NO** |
| --- | --- | --- |
| **Tingling** |  |  |
| **Tingling** |  |  |
| **Itching** |  |  |
| **Numbness** |  |  |

- **In general, is the pain caused or increased by:**

|  | **YES** | **NO** |
| --- | --- | --- |
| **Rubbing on the painful area** |  |  |
| **Pressure on the painful area** |  |  |
| **Contact with a cold object on the painful area** |  |  |
| **Contact with a hot object on the painful area** |  |  |

- **Does your pain meet one or more of the following criteria:**

|  | **YES** | **NO** |
| --- | --- | --- |
| **It was one of the first symptoms of Parkinson's disease** |  |  |
| **It is on the side most affected by Parkinson's disease** |  |  |
| **She Increases when your state Engine worsens** |  |  |
| **It is improved by taking antiparkinsonian drugs** |  |  |
| **It is present at night** |  |  |
| **It is present diffusely on your body** |  |  |
| **It moves from one place to another in your body** |  |  |

| **MDQ UPDRS PART III: ENGINE REVIEW**    Overview: This part of the scale assesses the motor signs of Parkinson's disease. When administering Part III of the MDS-UPDRS, the investigator should adhere to the following guidelines:  At the top of the form, note if the patient is on medication to treat Parkinson's symptoms and, if on levodopa, when was the last dose.    Similarly, if the patient is receiving medication to treat the symptoms of  Parkinson's, record the patient's clinical condition using the following definitions:  ON: Typical functional status when patients take medical treatment and respond well. OFF: Typical functional status when patients have a poor response despite taking their medications.    The investigator will have to: "evaluate what you see". It is generally accepted that concurrent medical problems such as stroke, paralysis, osteoarthritis, contracture, and orthopedic problems such as hip or knee replacement, and scoliosis, can interfere with individual items in motor examination. In situations where it is absolutely impossible to assess (amputations, paralysis, limb in a cast) use the NE notation for Not Evaluable. Otherwise, evaluate the performance of each task as the patient performs it in the context of co-morbidities.    All items must have a full grade (no half point, no missing score).    Specific instructions are given for the evaluation of each item. They will have to be followed in all cases. The investigator demonstrates as he or she describes the tasks that the patient must perform, and assesses the function immediately afterwards. For global spontaneous movement and resting tremor items (3.14 and 3.17), these items were deliberately placed at the end of the scale because the score-appropriate clinical information will be obtained from the entire examination.    At the end of the assessment, indicate whether dyskinesia (chorea or dystonia) was present at the time of the examination, and if this was the case if these movements interfered with the motor examination. |  |
| --- | --- |
| **3a** Is the patient receiving medication to treat the symptoms of Parkinson's disease?   NO  YES |  |
| **3b** If the patient is receiving medication to treat the symptoms of Parkinson's disease, record the patient's clinical status using the following definitions:   ON. ON corresponds to the typical functional status when patients receive their medication(s) and have a good response.   OFF. OFF is the typical functional status when patients have a poor response despite taking medication. |  |
| **3c** Is the patient on levodopa?   NO  YES  3.c1 If yes, minutes since last dose of levodopa: ______ |  |
| **3.1 SPEECH** |  |

| Instructions to the Investigator : Listen to the patient's free, fluent speech and engage in conversation if necessary. Suggested topics: Find out about the patient's job, favourite hobbies, exercises or how they got to the doctor's office? Evaluate volume, modulation (prosody), clarity, including articulation, palilalia (repetition of syllables), and tachyphemia (rapid speech linking syllables together). | | |  |
| --- | --- | --- | --- |
| 1. :Normal: 2. :Minimal: 3. :Light: 4. :Moderate: 5. :Severe: | | No speech problem.    Loss of modulation, diction or volume, but all words are still easy to understand.    Loss of modulation, diction or volume with a few unclear words but the majority of sentences are easy to follow.    The speech is so difficult to understand that some, but not most, sentences are misunderstood.    Most speech is difficult to understand or unintelligible. | □ |
| **3.2 FACIAL EXPRESSION**    Instructions to the Investigator : Observe the patient sitting, resting for 10 seconds, without speaking and also while speaking. Observe the frequency of blinking, face mask or loss of facial expression, spontaneous smile and lip spacing.      0: Normal: Normal facial expression. | | |  |
| 1: Minimal: | Minimal masked facies, only manifested by a decrease in the frequency of blinking. | |  |
| 2: Lightweight: | In addition to the decrease in the frequency of blinking, the facies in | |  |
| 1. :Moderate: 2. :Severe: | Mask is present in the lower part of the face, i.e. scarcity of movements around the mouth, such as a decrease in spontaneous smile, but the lips are not separated.    Face in mask with sometimes the lips separated when the mouth is at rest.    Face mask with lips separated most of the time when the mouth is at rest. | | □ |
| **3.3 RIGIDITY**    Instructions to the Investigator | : Rigidity is judged on slow and passive movements of the | | **Neck**  □ |

| simultaneously. If no r  since you are testing       1. :Normal: 2. :Minimal: 3. :Light: 4. :Moderate: 5. :Severe: | major joints, when the patient is in a relaxed position and the investigator manipulates the patient's limbs and neck. First, test without activation maneuver, test and evaluate the neck and each limb separately. For the arms, test the wrist and elbow joints simultaneously. For the legs, test the hip and knee joints  igidity is detected, use an activation maneuver (from  sensitization) such as tapping the fingers, opening and closing the fist, or tapping the heel, on a limb that is not being tested. Explain to the patient to be as relaxed as possible from rigidity.  No rigidity.    Rigidity only detected with the activation maneuver.    Stiffness detected without activation maneuver, but full amplitude of mobilization is easily performed.    Rigidity detected without activation maneuvers; the full amplitude of mobilization is carried out with effort.    Rigidity detected without activation maneuver and full amplitude of mobilization is not achieved. | **ESD**  □  **ESG**  □  **EID**  □  **EIG** □ |
| --- | --- | --- |
| **3.4 FINGER TAPPING**    Instructions to the Investigator : Each hand is tested separately. Demonstrate the task, but do not continue to perform the task while the patient is being tested. Instruct the patient to tap the index finger on the thumb 10 times as quickly and amply as possible. Evaluate each side separately by assessing speed, amplitude, hesitations, halts and decrease in amplitude. | |  |
| 0: Normal: | No problem. |  |
| 1: Minimal: | Any of the following situations: a) the regular rhythm is |  |
| 1. :Light: 2. :Moderate: | broken by one or two interruptions or hesitations in the tapping movement; b) minimal slowdown; c) the amplitude decreases near the end of the 10 taps.    Any of the following: a) 3 to 5 interruptions during tapping; b) slight slowdown; c) the amplitude decreases halfway through the sequence of 10 taps    Any of the following: a) more than 5 interruptions during tapping or at least one prolonged pause in the current movement; b) moderate slowdown; c) the amplitude decreases starting from the first tap. | **ED**  □    **EG**  □ |

| 4: Severe: Cannot or can only barely perform the task because of slowdown, interruptions, or decreases in amplitude. |  |
| --- | --- |
| **3.5 HAND MOVEMENTS**    Instructions to the Investigator : Test each hand separately. Demonstrate the task but do not continue to perform the task while the patient is being tested. Instruct the patient to close the fist, elbow bent, so that the palm is facing the investigator. Ask the patient to open their hand 10 times as wide and as quickly as possible. If the patient is unable to clench their fist or open their hand wide, remind them to do so. Evaluate each side separately, evaluating speed, amplitude, hesitations, halts, and amplitude decrease.       1. :Normal: No problem.      1. : Minimal: Any of the following situations: a) the regular rhythm is broken by one or two interruptions or hesitations in the movement; b) minimal slowdown; c) the amplitude decreases near the end of the task.      1. :Light: Any of the following: a) 3 to 5 interruptions   during movements; b) slight slowdown; c) the amplitude decreases to half of the task.     1. : Moderate: Any of the following: a) more than 5 interruptions during the movement or at least one prolonged stop (blockage) in the current movement; b) moderate slowdown; c) the amplitude decreases from the first opening and closing sequence.      1. : Severe: Cannot or can only barely perform the task because of slowdown, interruptions, or decreases in amplitude.       **3.6 PRONATION AND SUPINATION OF THE HANDS**      Instructions to the Investigator : Test each hand separately. Demonstrate the task, but do not continue to perform the task while the patient is being tested. Instruct the patient to extend the arm in front of their body, palm down, and then turn the palms up and down, alternately 10 times as quickly and amply as possible.  Evaluate each side separately by evaluating speed, amplitude, hesitations, halts, and amplitude decrease.       1. :Normal: No problem.      1. :Minimal: Any of the following situations: a) the regular rhythm is | **ED**  □    **EG**  □ |

| 1. :Light: 2. :Moderate: 3. :Severe: | broken by one or two interruptions or hesitations of movement; b) minimal slowdown; c) the amplitude decreases near the end of the sequence.    Any of the following: a) 3 to 5 interruptions during movements; b) slight slowdown; c) the amplitude decreases to half of the sequence.    Any of the following: (a) more than 5 interruptions during the movement or at least one prolonged stop (blockage) in the current movement; b) moderate slowdown; c) the amplitude decreases starting from the first prono-supination sequence.    Cannot or can only barely perform the task because of slowdown, interruptions, or decreases in amplitude. | **ED**  □        **EG**  □ |
| --- | --- | --- |
| **3.7 TOE TAT**    Instructions to the Investigator : Place the patient seated in a chair with a straight back and armrests with both feet on the floor. Test each foot separately. Demonstrate the task, but do not continue to perform the task while the patient is being tested. Instruct the patient to put the heel on the floor in a comfortable position, then tap the toes 10 times as widely and quickly as possible. Evaluate each side separately by evaluating speed, amplitude, hesitations, halts, and amplitude decrease.      0: Normal: No problem. | |  |
| 1: Minimal: | Any of the following situations: a) the regular rhythm is |  |
| 1. :Light: 2. :Moderate: 3. :Severe: | broken by one or two interruptions or hesitations in the tapping movement; b) minimal slowdown; c) the amplitude decreases near the end of the 10 taps.    Any of the following: a) 3 to 5 interruptions during tapping motions; b) slight slowdown; c) the amplitude decreases to half of the task.    Any of the following: a) more than 5 interruptions during the tapping movements or at least one prolonged stop (blockage) in the current movement; b) moderate slowdown; c) the amplitude decreases after the first tapping.    Cannot or can only barely perform the task because of slowdown, interruptions, or decreases in amplitude. | **ED**  □    **EG**  □ |
| **3.8 LEG AGILITY** | |  |

| Instructions to the investigator separately. Do the dice         1. :Normal: 2. :Minimal: 3. :Light: 4. :Moderate: 5. :Severe: | : Seat the patient in a straight-back chair and  Armrests. The patient should have both feet comfortably on the ground. Test each leg showing the task, but don't continue to perform the task  while the patient is being tested. Instruct the patient to put the foot on the ground in a comfortable position and then lift and tap the foot on the ground 10 times as high and quickly as possible. Evaluate each side separately by evaluating speed, amplitude, hesitations, halts, and amplitude decrease.  No problem.    Any of the following situations: a) the regular rhythm is broken by one or two interruptions or hesitations in the movement, b) minimal slowing down; c) the amplitude decreases towards the end of the task.    Any of the following: a) 3 to 5 interruptions during movements; b) slight slowdown; c) the amplitude decreases to half of the task.    Any of the following: (a) more than 5 interruptions during the movement or at least one prolonged stop (blockage) in the current movement; b) moderate slowing of speed; c) the amplitude decreases after the first tapping.    Cannot or can only barely perform the task because of slowdown, interruptions, or decreases in amplitude. | **ED**  □    **EG**  □ |
| --- | --- | --- |
| **3.9 LIFTING THE CHAIR**    Instructions to the Investigator : Place the patient seated in a chair with a straight back and armrests, with both feet on the floor and firmly seated in the chair (if the patient is not too short). Ask the patient to cross their arms over their chest and then stand up. If the patient is unsuccessful, repeat this trial up to a maximum of 2 times more. If the patient is still unsuccessful, allow the patient to step to the edge of the chair to stand up with their arms crossed over their chest. Allow only one trial in this position. If he is unsuccessful, allow the patient to lift himself up using his hands on the armrests of the chair. Allow a maximum of 3 push attempts. If it still does not succeed, help the patient to stand up. After the patient is standing, observe the posture for item 3.13.       1. :Normal: No problem. Able to get up quickly without hesitation.      1. : Minimal: Rising is slower than normal; or may have required more than one attempt; or may need to step forward to the edge of the chair to get up.   No need to use the armrests of the chair. | | □ |

| 1. :Light: Gets up using the armrests of the chair without difficulty.      1. :Moderate: Needs help to get up, but tends to fall backwards; or   may have to try more than once using the armrests of the chair, but can stand up without assistance.     1. :Severe: Unable to get up without help. | |  |
| --- | --- | --- |
| **3.10 WALKING**    Instructions to the Investigator : Gait is best assessed by having the patient walk away and then back to the investigator so that both the right and left sides of the body can be easily observed simultaneously. The patient will be required to walk at least 10 m and then turn and return to the investigator. This item measures multiple behaviors: stride amplitude, stride speed, height at which the foot is lifted, heel strike while walking, pivoting, and arm swing, but not freezing. Also evaluate for "freezing" (next item 3.11) while the patient is walking. Observe the posture for item 3.13.    0: Normal: No problem. | |  |
| 1: Minimal: | Autonomous ambulation with minimal impairment of gait. |  |
| 1. :Light: 2. :Moderate: 3. :Severe: | Autonomous ambulation but with a substantial impairment in gait.    Requires an assistive device for safe walking (cane, walker), but no human assistance.    Can't walk at all or only with the help of another person. | □ |
| **3.11 FREEZING**    Instructions to the investigator : During gait assessment, also assess for episodes of gait blockage (freezing). Observe hesitation when starting and trampling movements, especially when turning around and reaching the end of the task. To the extent permitted by their safety, patients may not use sensory tricks during the evaluation.     1. :Normal: No blockage.      1. : Minimal: Locks on start-up, U-turn, or when crossing the threshold of a door with a single stop during any of these events, but then continues in a united manner without jamming during line operation | | □ |

|  | right. |  |
| --- | --- | --- |
| 2: Lightweight: | Locks when starting, turning, or crossing a doorway with more than one stop during any of these activities, but continues in a smooth manner without jamming when walking in a straight line. |  |
| 3: Moderate: | Locks once when walking in a straight line. |  |
| 4: Severe: | Locks up several times when walking in a straight line. |  |
| **3.12 POSTURAL STABILITY**    Instructions to the Investigator : The test examines the response to a sudden displacement of the body produced by a rapid, forceful push on the shoulders, while the patient is standing with their eyes open, feet comfortably separated and parallel. Retropulsion test. Stand behind the patient and tell them what is going to happen. Explain to him or her that he or she is allowed to take a step back to avoid falling. There should be a solid wall behind the examiner, at least 1 or 2 m away, to allow observation of the number of retropulsive steps. The first push is an instructional demonstration and is intentionally lighter and unevaluated. The second time, the shoulders are pulled abruptly and forcefully toward the investigator with enough force to shift the center of gravity such that the patient MUST take a step back. The investigator needs to be close to catch up with the patient, but must stand back enough to leave enough room for the patient to take several steps and regain balance.  Do not allow the patient to bend the body abnormally forward to anticipate the impact. Observe the number of steps backwards or the fall. The test is considered normal as long as the patient does not take more than 2 steps (including 2 steps) backwards to regain his balance, so that abnormal assessments start from 3 steps. If the patient does not understand the test, the investigator may repeat the test so that the assessment is in line with an estimate that the investigator considers to reflect the patient's limitations, rather than the patient's misunderstanding of the test or lack of preparation. Observe the standing posture for item 3.13. | | □ |
| 0: Normal: | No problem: Regain your balance with 1 or 2 steps. |  |
| 1: Minimal: | 3 to 5 steps, but the subject regains his balance without help. |  |
| 2: Lightweight: | More than 5 steps, but the subject regains his balance without help. |  |
| 3: Moderate: | Keeps the balance standing securely, but with no postural response; fall if he is not caught by the examiner. |  |
| 4: Severe: | Very unstable, tends to lose balance spontaneously or just with a slight push on the shoulders. |  |
|  |  |  |

| **3.13 POSTURE**    Instructions to the Investigator  You notice poor posture, improve (see option 2 below. Observe the       1. :Normal: 2. :Minimal: 3. :Light: 4. :Moderate: 5. :Severe: | : Posture is assessed while the patient is standing after  having risen from a chair, during walking, and during the examination of postural reflexes. If tell the patient to stand up straight and see if the posture  -below). Rate the worst posture observed in these 3  flexion and the tendency to lean to the sides.  No problem.    Doesn't stand quite straight, but posture could be considered normal for an older person.    Indisputable flexion, scoliosis or latero-deviation, but the patient can correct the posture to a normal posture when asked to do so.  Flexed posture, scoliosis or latero-deviation that cannot be corrected voluntarily to a normal posture by the patient.    Bending, scoliosis, or leaning attitude with an extremely abnormal posture. | □ |
| --- | --- | --- |
| **3.14 OVERALL SPONTANEITY OF MOVEMENT (body bradykinesia)**    Instructions to the Investigator : This comprehensive assessment combines all observations on slowness, hesitation, reduced range of motion, and scarcity of movement in general, including reduced movement and crossing of the legs. This observation is based on the investigator's overall impression after observing spontaneous gestures in a sitting position, and the nature of getting up and walking. | |  |
| 0: Normal: | No problem. |  |
| 1: Minimal: | Overall slowness and poverty of minimal spontaneous movements. |  |
| 1. :Light: 2. :Moderate: 3. :Severe: | Overall slowness and poverty of light spontaneous movements.    Overall slowness and poverty of moderate spontaneous movements.    Overall slowness and poverty of severe spontaneous movements. | □ |
| **3.15 POSTURAL TREMOR OF THE HANDS**    Instructions to the Investigator : Any tremor, including the re-emergence of the resting tremor present in this posture, should be included in this evaluation. Odds each hand | | **ED** |

| comfortably seconds.       1. :Normal: 2. :Minimal: 3. :Light: 4. :Moderate: 5. :Severe: | separately. Rate the greatest amplitude observed. Instruct the patient to extend their arms in front of their body, palms facing down. The wrist should be straight and the fingers trimmed so that they do not touch each other. Observe this pose for 10  No trembling.    The tremor is present but has less than 1 cm in amplitude.    The tremor has at least 1, but no more than 3 cm in amplitude.    The tremor has at least 3 but no more than 10 cm in amplitude.    The tremor has at least 10 cm in amplitude. | □    **EG**  □ |
| --- | --- | --- |
| **3.16 HAND ACTION TREMOR**    Instructions to the Investigator : This test is based on the finger-nose maneuver. With the arm starting with the position extended, have the patient perform at least 3 finger-nose maneuvers with each hand, reaching as far as possible to the investigator's finger. The finger-nose maneuver should be performed slowly enough not to hide any tremors that can occur with very rapid arm movements. Repeat with the other hand, evaluating each hand separately. The tremor may be present through movement or while it has reached one or the other target (nose or finger). Evaluate the greatest amplitude observed.    0: Normal: No trembling. | |  |
| 1: Minimal: | The tremor is present but less than 1 cm in amplitude. |  |
| 1. :Light: 2. :Moderate: 3. :Severe: | The tremor has at least equal to 1 but not more than 3 cm in amplitude.    The tremor has at least 3 but not more than 10 cm in amplitude.    The tremor has at least 10 cm in amplitude. | □ |
| **3.17 AMPLITUDE** | **OF THE RESTING TREMOR** |  |
| Instructions to the Investigator : This item and the following item were intentionally placed at the end of the examination to allow the evaluator to gather observations of the resting tremor that may appear at any time during the examination, including the patient's quiet sitting attitude, walking, and all activities during which certain parts of the body are in motion, but others are at rest. Rate the maximum amplitude observed at any given time as the final score. Rate only the amplitude and not the persistence or intermittency of the tremor. | | **ESD**  □  **ESG**  □  **EID**  □  **EIG** □ |

| As part of this assessment, the patient should sit quietly in an armchair with their hands on the armrests (not their knees), and their feet comfortably on the floor for 10 seconds without any further instructions. The resting tremor is evaluated separately for the 4 limbs and also for the lip/jaw assembly. Evaluate only the maximum amplitude observed at any given time as a final evaluation.    *Extremity Assessment* :  0: Normal: No trembling. | |  |
| --- | --- | --- |
| 1: Minimal: | < 1 cm in maximum amplitude. |  |
| 1. :Light: 2. :Moderate: 3. :Severe:       *Evaluation for l* 0: Normal:   1. :Minimal: 2. :Light: 3. :Moderate: 4. :Severe: | > to 1 cm but < to 3 cm in maximum amplitude.    3 to 10 cm in maximum amplitude.    > to 10 cm in maximum amplitude.    *Fever/jaw:* no tremor.    < 1 cm in maximum amplitude.    > to 1 cm but < to 2 cm in maximum amplitude.    < 2 cm but at > to 3 cm in maximum amplitude.    > to 3 cm in maximum amplitude. | **Lips, jawline**  □ |
| **3.18 CONSTANC**    Instructions to the rest and focuses when the different the examination of such squotation.       1. :Normal: 2. :Minimal: 3. :Light: | **E OF RESTING TREMOR**  Stigator : This item receives a single evaluation for all the tremor of the resting tremor during the period of the examination  parts of the body are at rest. It is deliberately stated at the end of the article that several minutes of information can be grouped together in the  No trembling.    Resting tremor is present, less than 25% of the entire period of the examination.    Resting tremor is present, between 26-50% of the entire period of the examination. | □ |
| 3: Moderate: | Resting tremor is present, between 51 and 75% of the entire period of the examination. |  |
| 4: Severe: | Resting tremor is present, more than 75% of the entire period of the examination. |  |
| **RESONISSEME** | **NT OF DYSKINESIAS ON THE EVALUATIONS OF THE PART** |  |
| **III**     1. Were dyskinesias (chorea or dystonia) present during the examination? NO YES 2. If so, did these movements interfere with your assessments?   NO YES      **HOEHN & YAHR LADDER**   1. :Asymptomatic 2. : Unilateral involvement only 3. : Bilateral involvement without loss of balance 4. : Mild to moderate involvement; postural instability but physically autonomous; Needs assistance to recover from the impulse test. 5. : Severe disability; still able to walk or stand without assistance. 6. : Forced to be in a wheelchair or bedridden unless helped. | | □ |

### HAD Scale

**Circle your answer:**

**Has. I feel tense or angry:**

3. Most of the time

2. Often

1. From time to time

0. Never

**D. I enjoy the same things as before:**

1. Yes, just as much
2. Not as much
3. Only a little
4. Almost more

**Has. I have a feeling of fear as if something horrible is going to happen to me:**

3. Yes, very clearly

2. Yes, but it's not too serious

1. A little, but that doesn't worry me

0. Not at all

**D. I laugh easily and see the bright side of things:**

1. As much as in the past
2. Not as much as before
3. Really less than before
4. Not at all

**Has. I'm worried:**

3. Very often

2. Quite often

1. Occasionally

0. Very occasionally

**D. I'm in a good mood:**

3. Never

2. Rarely

1. Quite often

0. Most of the time

**Has. I can sit quietly and do nothing and feel relaxed:**

1. Yes, whatever happens
2. Yes, in general
3. Rarely
4. Never

**D. I feel like I'm running in slow motion:**

3. Almost always

2. Very often

1. Sometimes

0. Never

**Has. I feel scared and my stomach is in knots:**

1. Never
2. Sometimes
3. Quite often
4. Very often

**D. I'm no longer interested in my appearance:**

3. Not at all

2. I don't pay as much attention to it as I should

1. I may not pay as much attention to it anymore

0. I pay as much attention to it as I did in the past

**Has. I'm on the move and can't stay still:**

3. Yes, it is absolutely the case

2. A little

1. Not so much

0. Not at all

**D. I'm looking forward to doing a few things:**

1. As much as before
2. A little less than before
3. Much less than before
4. Hardly ever

**Has. I experience sudden feelings of panic:**

3. Really, very often

2. Quite often

1. Not very often

0. Never

**D. I can enjoy a good book or a good radio or television program:**

1. Often
2. Sometimes
3. Rarely
4. Very rarely

**Total A (anxiety): ________/21 Total D (Depression) :________/21**

**TOTAL SCORE = ________**

**Total A or D**

0 - 7 Normal

- 1. - 8 Moderate
  2. – 14 Medium

15 - 21 Severe

1. Evaluation of the pain perception threshold performed before and after the PR or MS session [↑](#footnote-ref-1)
