# Supplementary material for: Effect of foot reflexology on chronic pain in Parkinson’s disease: A randomized controlled trial
Source: PLoS One. 2025 Jul 28;20(7):e0327865. doi: 10.1371/journal.pone.0327865 (PMC12303304; doi:10.1371/journal.pone.0327865)
Supplement: Attachment — Submitted filename: Response to reviewers.docx [file pone.0327865.s011.docx]

**We thank the editors and reviewers for their relevant interrogations and comments.**

**Line numbers refer to the file with tracked changes.**

**Editors:**

1. Please ensure that your manuscript meets PLOS ONE's style requirements, including those for file naming. The PLOS ONE style templates can be found at https://journals.plos.org/plosone/s/file?id=wjVg/PLOSOne_formatting_sample_main_body.pdf and https://journals.plos.org/plosone/s/file?id=ba62/PLOSOne_formatting_sample_title_authors_affiliations.pdf.

**As requested, the files names have been modified according to your policies as well as the format of the affiliations and the main body.**

2. In the online submission form, you indicated that [The data that support the findings of this study are available in the Supporting Information files. The raw MRI data are available on reasonable request to the corresponding author. The protocol is available on the journal webpage of this article.].

All PLOS journals now require all data underlying the findings described in their manuscript to be freely available to other researchers, either 1. In a public repository, 2. Within the manuscript itself, or 3. Uploaded as supplementary information.

This policy applies to all data except where public deposition would breach compliance with the protocol approved by your research ethics board. If your data cannot be made publicly available for ethical or legal reasons (e.g., public availability would compromise patient privacy), please explain your reasons on resubmission and your exemption request will be escalated for approval.

**Raw MRI data are considered medical information in Europe. As the protocol did not explicitly stated that the data would be made available, we propose, for ethical or legal reasons, the alternative of providing them upon request. Additionally, we have already shared connectivity values in “S1 Data. Data supporting this article”.**

3.  Please include captions for your Supporting Information files at the end of your manuscript, and update any in-text citations to match accordingly. Please see our Supporting Information guidelines for more information: <http://journals.plos.org/plosone/s/supporting-information>.

**The captions of the supporting information files have been added to the manuscript lines 657-727). We renamed the files accordingly.**

**Reviewer #1:** It appears that all the comparative analysis in the primary is simple and done correctly with the correct conclusions being drawn from the data. However, the design and analysis need a more formal development.
It looks as if the sample size is based on a parametric assumption and some of the analysis is done nonparametrically which makes sense given the small sample. The sample size needs further justification.

For the design what is the rationale for the 20 mm difference and 15 sd. and what reference is being used to make the calculation? There are many analyses done with a limited sample. One should be aware of possible multiple comparison issues.

**Due to the particular nature of the data and the limited sample size, we have chosen to process the clinical data with non-parametric assumptions. The connectivity values however were perfectly parametric and processed accordingly. We believe we have limited the number of analyses to the bare minimum considering our objectives.**

**Based on a previous study in PD patients (Nebe A, Ebersbach G. Pain intensity on and off levodopa in patients with Parkinson’s disease. Mov Disord 2009;24:1233–1237), we assumed an expected difference between reflexology and Sham massage of 20 mm and and a standard deviation of 15.0mm. In addition, a difference from 10 to 20 mm is usually considered as the clinically important difference between analgesic treatment and placebo (Dworkin RH, Turk DC, McDermott MP, et al. Interpreting the clinical importance of group differences in chronic pain clinical trials: IMMPACT recommendations. Pain 2009;146:238–244).**

**For the design, additional information can be found in Figure 1 of the Supplementary information. Due to the journal’s policy, the design figure could not be loaded as Figure 1. We believe that we have provided all the mandatory elements in the design section according to the CONSORT.**

The secondary and exploratory analysis is interesting and appears to have been well executed, However, some detail is needed for clarification. How was the general linear model implemented? Clustering and multivariate statistics are involved. One reads the Annex and Figure 2 and notes some detail and the clustering. However, what other multivariate statistics are used and how?

***In Seed-to-Voxel.* In the first level analysis, the seed’s activation time course is correlated to the activation time courses of every voxel in the brain. In the second level analysis, a general linear model is automatically computed by the CONN software depending on the study design and the selected statistical contrast (in our case, a 2x2 repeated measures ANOVA). The voxels that are above the statistical threshold are grouped into clusters. The clusters sizes are corrected using the Gaussian Random Field Theory.**

***In ROI-to-ROI.* In the first model analysis, the activation time course of each ROI is correlated to every other by pairs. In the second model analysis, clusters of ROIs are formed using a hierarchical clustering method based on ROI-to-ROI anatomical proximity and functional similarity metrics. Then the Functional Network Connectivity (FNC) method evaluate the within- and between-clusters functional connectivity using a general linear model (Jaffri, 2008).**

**We processed the second level analyses with the default method in CONN because we had no a priori on the estimated effect size.**

**The term “multivariate statistics” refers to the statistical contrast implemented in the second level analysis. Here, it is the 2x2 repeated measures ANOVA which belongs to multivariate statistical analyses. In CONN, it is also possible to adjust with covariables, but we have chosen not to adjust our analyses due to the limited sample size and a lack of statistical power.**

**Reviewer #2:** 1、 The authors investigated the effects of foot reflexology on chronic pain in patient with Parkinson's disease and associated changes in brain functional connectivity. The methodology mentions that "The dopaminergic medication as well as antalgic treatment had to remain stable for at least 4 weeks before and throughout the study." However, specific details about baseline antalgic treatment (e.g., whether participants were taking antalgic medicine and medication history, types, and dosages) were not provided. Considering the potential impact of antalgic on intervention outcomes, this information should be clarified and analyzed.

**Only few patients were receiving analgesic treatment, and among those who did, it was mostly paracetamol taken irregularly as they deem necessary to relieve their pain. Therefore, we could not include this parameter as covariate neither analyze the dose as a clinical variable. This was added to the limits (lines 492-494).**

2、In the study design, participants received one 60-minute FR or SM intervention every three weeks, totaling four sessions. Could the relatively low frequency of interventions have influenced the effectiveness of the treatment? This possibility should be addressed.

**The specific reflexology protocol used in the present study was developed in collaboration with experienced reflexology practitioners. It reflects the reality of reflexology practice, considering the body's response, which typically occurs within three weeks for chronic conditions (Faure Anderson, 2018, EAN. 9782813217707). However, the peak effect varies between individuals and remains uncertain. The protocol was validated by the working group on reflexology evaluation (Descamps et al, 2025, DOI : 10.3917/heg.114.0356).**

**In the context of chronic conditions, the literature generally supports a frequency of one session every three weeks to one month, suggesting that our intervention frequency aligns with current practice. However, regarding the optimal timing for outcome measurement, we had no strong prior assumptions. We therefore selected a three-week interval, consistent with a chronic care framework.**

3、The study included participants with various types of pain, but no subgroup analysis based on pain type or stratification by pain severity was performed. This lack of detailed analysis makes the study design appear somewhat simplistic.

**We agree that the interventions could not have the same effects depending of the type of pain. We did not have a prior assumption in which type of pain could be better ameliorated by FR. That is why we included all types of pain in PD. We did not process subgroup analyses because of our limited sample size (n=15 in each group) and a lack of statistical power due to the heterogeneity of the data, but we agree that it would be important to address this in a future study.**

4、The baseline data show that the duration of pain symptoms among participants is longer than the duration of their PD. Is this finding reasonable? The authors should provide an explanation for this observation.

**Pain could be a prodromal feature of Parkinson’s disease and can appears years before the motor symptoms leading to the diagnosis (Buhman, 2020, doi: 10.1007/s00415-017-8426-y), so this finding is coherent with clinical and in research observations.**

**Reviewer #3:** The current study examined the therapeutic effect of foot reflexology (FR) in Parkinson’s disease (PD) patients with chronic pain using a randomized controlled trial, in which the therapeutic effects were assessed from both behavioral and neuroimaging perspectives. However, the results showed that there was no significant therapeutic effect of FR compared to sham massage (SM). The description of the significance and feasibility of fMRI analysis and ROI selection in the current study needs to be improved. Here are some of my specific comments:

Abstract
1. Authors should reorganize the Abstract section to comply with the formatting requirements of journal.

**Plos One requests to comply with the CONSORT checklist for an RCT. For the abstract, we believed that we provided all necessary items in that checklist, and we added some details in the methods and results.**

Introduction
1. The second paragraph of the Introduction should be switched with the third paragraph, and the second paragraph is too simplistic, with insufficient introduction of the significance and feasibility of why the insula, the nucleus accumbens, and the mPFC were chosen as ROIs. In addition, the feasibility and significance of ROI-to-ROI analyses are missing.

**The second paragraph of the introduction have been switched with the third paragraph.**

**For the Seed-to-Voxels analyses, these 3 areas were chosen as seeds because they have shown altered connectivity in pain conditions (Tu et al., 2019; Baliki et al., 2006, 2012) and in PD pain (Polli et al., 2016; Kinugawa et al., 2022, Joineau et al., 2024). Studies focusing on chronic pain in PD and using functional connectivity as outcome are very limited.**

**The main and the most studied networks implicated in these processes are the default mode network (DMN), the ECN and the Salience Network (SN) (Shirer et al., 2012; Richiardi et al., 2015).**

**The sensory motor network (SMN) is also implicated in attentional processes, involved in tactile touch and pain (Shirer et al., 2012; Richiardi et al., 2015).**

**Finally, we wanted to explore a new network specifically implicated in pain, the neural network correlates of pain (NNCP) from Descamps et al., 2023.**

**More details about the selection of seeds and networks were given in the introduction (lines 96-104) and method sections (lines 269-272).**

2. The last two paragraphs of the introduction could be merged.
**The last two paragraphs regarding the objectives were merged.**

Methods
1. The present treatment protocol for FR comprises four FR treatments, each with a duration of one hour, administered with a three-week interval between sessions. How was this protocol determined?

**The specific reflexology protocol used in the present study was developed in collaboration with experienced reflexology practitioners. It reflects the reality of reflexology practice and has been subsequently validated by this reflexology evaluation working group (Descamps et al, GER: a Think and Do Tank for the assessment of reflexology, HEGEL, Vol. 15 N°1 - 2025).**

2. Why is the pain threshold measured using thermal stimulation?

**The nociceptive pain threshold was measured using thermal stimulation because it is one of the common methods to assess this parameter, alongside cold, pressure, and electrical stimulations. Parkinson’s disease patients have a lower nociceptive heat threshold compared to healthy subjects (reviewed by Sung et al., 2017, doi: 10.1016/j.parkreldis.2017.12.031). We expected that FR could normalize it which was not the case.**

3. In section of Seed-to-Voxel functional connectivity, the coordinates of the mPFC and the radius of all seed need to be reported.

**The coordinates of all the seeds have been added as well as the radius size of the insulae and mPFC (lines 256-262). For the nuclei accumbens, the segmentation was implemented in the CONN software and do not have the shape of a sphere.**

**We have chosen not to use the default atlas for the insulae and the mPFC as we wanted to separate the anterior and posterior parts of the insulae and because the mPFC is not well defined in the default Harvard-Oxford atlas directly implemented in CONN.**

4. The description of the ROI-to-ROI analysis is not clear, why these networks were chosen is not presented in the Introduction or Methods. And how were these networks obtained, was it through ICA? Finally, did the ROI-to-ROI analysis also use GRF correction?

**We thank the reviewer for this question which provides an opportunity to further clarify the criteria for networks selection.**

**In the introduction, we have added a paragraph showing that PD patients have an alteration of networks associated with attentional processes such as the executive control network (ECN) (Tan et al., 2015) (lines 94-106).**

**The main and the most studied networks implicated in these processes are the default mode network (DMN), the ECN and the Salience Network (SN) (Shirer et al., 2012; Richiardi et al., 2015).**

**The sensory motor network (SMN) is also implicated in attentional processes, involved in tactile touch and pain (Shirer et al., 2012; Richiardi et al., 2015).**

**Finally, we wanted to explore a new network specifically implicated in pain, the neural network correlates of pain (NNCP) from Descamps et al., 2023.**

**The DMN, SN, ECN and SMN were directly obtained from atlas files of Shirer et al., 2012; Richiardi et al., 2015. This atlas is often called the “Willard” atlas or the “Standford atlas”.**

**The NNCP was obtained from Descamps et al., 2023.**

**We did not perform any ICA or other analyses to obtain the networks, we used their respective atlas files.**

5. In first paragraph of section of 2.5 Statistical analyses, was it an estimate of the sample size? If so, what is the basis for estimate?

**The first paragraph of this section is indeed an estimation of the sample size needed for this study and with the VAS as first outcome.**

**Based on a previous study in PD patients (Nebe A, Ebersbach G. Pain intensity on and off levodopa in patients with Parkinson’s disease. Mov Disord 2009;24:1233–1237), we assumed an expected difference between reflexology and Sham massage of 20 mm and and a standard deviation of 15.0mm. In addition, a difference from 10 to 20 mm is usually considered as the clinically important difference between analgesic treatment and placebo (Dworkin RH, Turk DC, McDermott MP, et al. Interpreting the clinical importance of group differences in chronic pain clinical trials: IMMPACT recommendations. Pain 2009;146:238–244).**

6. The authors seem to have only examined the correlation between FC and VAS, and the authors might also consider examining whether there is a correlation between FC and KPPS or CPAQ-8.

**Since the main outcome of the study was the VAS, we decided to correlate this main outcome with the exploratory one: functional connectivity. We believed that a large number of correlation analyses would have drown out the interpretation of the results. However, the reviewer outlines an interesting point. Indeed, there is a correlation between the two connectivity changes of the nucleus accumbens and the KKPS and CPAQ8. However, the correlations seemed quite limited, and would not have pass a corrected threshold for multiple comparisons.**

**NA1 – KKPS Rho=-0.38; p=0.04**

**NA1 -CPAQ8 Rho=-0.41; p=0.03**

**NA2 – KPPS ; p > 0.05 (not significant)**

**NA2 – CPAQ8 Rho=-0.50; p=0.006**

**There is no significant correlation between the SMN (ROI-to-ROI) or mPFC (Seed-to-Voxels) connectivity changes and the KPPS and CPAQ8 changes (p>0.25).**

**The first two correlations (NA1 – KKPS and NA1 -CPAQ8) have a low Rho coefficient.**

**For the last correlation, the difference in the change of the pain acceptance between the two groups could be linked to different connectivity patterns between the nucleus accumbens and a cluster in the frontal medial cortex (Harvard Oxford CONN nomenclature). Both nucleus accumbens and frontal medial cortex are parts of the limbic system associated with pain acceptance. Nonetheless, the changes of CPAQ8 were very limited and do not reach a clinically significant difference. Therefore, we believe it should not be added in the manuscript.**

Results
1. Supplementary analyses should be added to the methods section. In addition, it is not clear why analyses were conducted using precentral gyrus, postcentral gyrus and supplementary motor area.

**As recommended, supplementary analyses were added to the method section (les 308-312).**

**These regions are entirely parts of the Sensori Motor Network (SMN) (Shirer et al., 2012; Richiardi et al., 2015). We have chosen to explore this network because it is implicated in pain and attentional processes. Moreover, since FR involves touch, we believed that the effect of this intervention in pain relief could be associated with changes in the SMN.**

**To detail this ROI-to-ROI analysis, the connectivity changes differences between the FR and SM groups were assessed for all pairs of ROIs in this SMN (as well as all the other networks presented). We implemented the second level analysis with a 2x2 repeated measures ANOVA.**

**There was no difference in the connectivity changes between the FR and SM group in the SMN.**

**However, there was a main effect of the “visit” factor without interaction with the group factor. There was a general decrease in connectivity for all patients in a cluster formed of the precentral gyrus, postcentral gyrus and supplementary motor area.**

**In ROI-to-ROI analysis, the connections between more than two areas can be grouped into clusters of connections if they share similar patterns of connectivity. The cluster found was composed of these three ROIs. Since the change of this connectivity was not correlated to the VAS diminution, it could not explain the FR and/or SM effects on pain relief.**

2. In Table 2, ‘*’ appears more appropriately after the P-value.

**“*” only assess the intragroup significant change and not the intergroup differences.**

3. Statistical values that appeared in the manuscript should be italicized, such as p.
**Statistical values have been italicized.**

Discussion
1. I disagree with the discussion in the third paragraph of Discussion, which categories FR as a form of manual therapy and suggests it is effective. However, the current study showed that there was no significant difference between FR and SM (placebo), which suggested that FR had no actual therapeutic effect.

**We hypothesized that FR would have a superior effect than SM on pain in PD and have chosen a control group with the same contextual effects and involving tactile touch as well.**

**FR did not show any significant effect compared to SM on pain in PD. Therefore, pressuring specific reflex zones do not have any clinical effect compared to tactile touch in general.**

**However, both FR and SM are considered manual therapy involving foot tactile touch. Therefore, since we witnessed pain diminution in both groups (significant in SM and tendency in FR), we think that foot tactile touch (touching the feet) could be effective in relieving pain in PD (of course linked to placebo effects).**

**Reviewer #4:** Title: Effect of foot reflexology on chronic pain in Parkinson’s disease: A randomized controlled trial (ID: PONE-D-25-02337)

The authors explored the effectiveness of Foot Reflexology (FR) and Sham Massage (SM) on PD patients with chronic pain. The VAS scores showed no significance between FR group and SM group, while the brain functional connectivity has some difference. This study provides new insights into PD. However, there are some issues that should be classified.
Abstract
1.It is suggested to improve the methods section in the Abstract.

**Plos One requests to comply with the CONSORT checklist for an RCT. For the abstract, we believed that we provided all necessary items in that checklist, and we added some details in the methods and results.**

2.More detailed results are better.

**We added some details to the results section of the abstract. We believe we have provided all the information needed in the CONSORT checklist.**

Introduction
3.More information on the pathophysiology of pain and studies of rs-fMRI on the brain function alterations in PD would be better.

**We wanted to focus on studies investigating pain in PD patients using rs-fMRI, but their number of publications is very low.**

4.It is recommended to integrate the last two paragraphs of Introduction.

**The last two paragraphs regarding the objectives were integrated.**

Methods
5.Why didn't this study set up a normal control group?

**In many Non-Pharmaceutical Interventions (NPI) protocols, the control group consisted of standard care. However, this design only allows us to see the global and contextual effects of the studied NPI. There are also active or passive controls in this type of trials (massage, music etc…). To study physical intervention, it is recommended to design a control group as close as possible to the intervention evaluated and involving the same contextual effects in order to isolate the specific ones (Hohenschurz-Schmidt D, et al., Recommendations for the development, implementation, and reporting of control interventions in efficacy and mechanistic trials of physical, psychological, and self-management therapies: the CoPPS Statement. BMJ. 2023 May 25;381:e072108. doi: 10.1136/bmj-2022-072108). It is why we thought that a classic foot massage could be closer to FR in terms of touch and interaction but without the specific reflex zones being pressured. We wanted to isolated the specific effects of pressuring these reflex zones from a general tactile stimulation and relaxation.**

**We also have discussed this choice of control group in the discussion (lines 497-509).**

6.What does adverse events usually mean?

**An adverse event is “an undesired effect of a drug or other type of treatment” (NIH). In Non-Pharmacological Interventions, adverse events are very rare but can involve pain, redness, cramps and allergic reactions. There were no adverse events in our study.**

7.In rs-fMRI analysis, why were the insula, the accumbens, and the mPFC chosen as ROI?

**These 3 areas were chosen as seeds because they showed altered connectivity in pain conditions (Tu et al., 2019; Baliki et al., 2006, 2012) and in PD pain (Polli et al., 2016; Kinugawa et al., 2022). Studies focusing on chronic pain in PD and using functional connectivity as outcome are very limited.**

8.It would be better to placed the specific coordinate points of mPFC and accumbens.

**The coordinates of all the seeds have been added as well as the radius size of the insulae and mPFC (lines 256-262). For the nuclei accumbens, the segmentation is implemented in the CONN software and do not have the shape of a sphere.**

**We have chosen not to use the default atlas for the insulae and the mPFC as we wanted to separate the anterior and posterior parts of the insulae and because the mPFC is not well defined in the default Harvard-Oxford atlas in CONN.**

9.What does NNCP refer to (line241)?

**We thank the reviewer for pointing out this oversight. The definition was added to the manuscript (line 273). NNCP means Neural Network Correlates of Pain (Descamps et al., 2023).**

10.For statistical analysis, in my opinion, the first paragraph regarding the sample size was not necessary.

**Plos One requests to comply with the CONSORT checklist for an RCT. The calculation of the sample size is one of the items.**

11.Bonferroni corrections method was used for multiple comparisons (line280), however, GRF method was mentioned before (line236), which one was it?

**In Seed-to-Voxel analyses, we have chosen the Gaussian Random Field Theory only to build the clusters of appropriate sizes in the second model analyses with the selected contrast. Then only the significant connectivity values were imported in Jamovi. For the ANOVAs post-hoc tests, we used Bonferroni corrections for intra-group changes. We used this particular method because CONN is not suited for post-hoc tests as a change in the contrast modifies the cluster size and connectivity values.**

**Another method would have been to build masks of significant clusters and assess post-hoc contrast directly in CONN between the seed and the clusters masks. However, this method does not consider the repetition of tests in the same connectivity values. Even if the cluster size is corrected, the repetition of tests in the same connectivity could still lead to false positive results.**

Discussion
12.The discussion regarding the changes of functional connectivity should be deeper.

**This is the first study focusing on the effects of FR in PD patients. Regarding functional connectivity, the findings remain very preliminary. The thalamus conveys pain-related information coming from the periphery to cortical regions, including the mPFC, which modulates attention and the emotional evaluation of pain through top-down control mechanisms (Schweinhartd and Bushell, 2010).**

**We hypothesized that the higher baseline connectivity observed in responders may reflect a more efficient top-down modulation of pain, thereby facilitating the treatment response. The decrease in connectivity in responders after treatment could illustrate a reduced compensatory mechanism, since the interventions are partly effective. In the opposite, the increase in connectivity in non-responders could represent a late compensatory mechanism that would be ineffective (added in lines 652-659).**

13.What are the possible reasons why FR was not particularly effective in patients with chronic pain in PD in this study, which differs from the results of previous studies?

**This study differs from previous studies with:**

**1- the choice of the control group, maybe it was too close to the studied intervention, (line 497).**

**2- the studied pathology: the effects of FR in PD pain have never been evaluated, (line 411).**

**3- the chronic aspect of pain: the studies with strong methodology only showed superior effect of FR compared to their control in acute pain. In chronic pain, they showed inconsistent results. (lines 419-427).**
